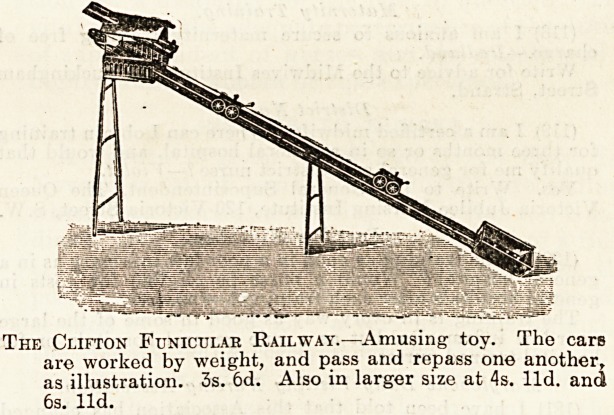# The Hospital. Nursing Section

**Published:** 1906-12-01

**Authors:** 


### The Hospital.

### Nursing Section.

Contributions for "THE HOSPITAL," should be addressed to the EDITOR, "THE HOSPITAL" NURSING SECTION, 28 & 29 Southampton Street, Strand, London, W.C.

NO. 1,054.—VOL. XLI SATURDAY, DECEMBER 1, 1906.

### Notes on News from the Nursing World.

### OUR CLOTHING DISTRIBUTION.

WE are now within a short time of the date when the whole of the contributions to our Christmas Distribution of clothing must be in hand. Articles should reach us not later than Monday, December 17, in order that they may be on view at the offices of THE HOSPITAL on Tuesday afternoon, the 18th, between 3 and 5 P.M. Since last week we have received parcels from Policy 5934 Pension Fund; Policy 101 Pension Fund; and "A Grimsby Nurse." All contributions should be addressed to the Editor, 28 and 29 Southampton Street, Strand, London, W.C., and should have "Clothing Distribution" written outside.

### THE NURSING DEPARTMENT AT THE WAR OFFICE.

WE are informed that in future all communications intended for the matron-in-chief of Queen Alexandra's Imperial Military Nursing Service should be addressed to her at the War Office, Whitehall, S.W. The Nursing Department in the splendid new building is on the third floor; and there is ample accommodation for meetings of the Advisory and Nursing Boards, for the matron-in-chief, who, as well as the principal matron, has her own private office, and for her clerical staff. One great convenience is a telephone in every room of the Department. Miss Keer and her staff only moved into their new quarters on Monday.

### ROYAL PORTSMOUTH HOSPITAL.

THE *Portsmouth Times* contains an account of an instructive discussion which took place at a meeting of the committee of management recently held, when the appointment of the assistant matron as matron, she having acted in the latter capacity for the last three months, came up for confirmation. It appears that when it was agreed that the assistant matron should act as matron for three months, it was distinctly laid down and understood, that this arrangement should have no bearing upon the final choice of a matron. Mr. Smyth and other governors strongly urged the desirability of advertising the post, disclaiming all personal feeling, and basing their contention upon public grounds, and the understanding arrived at previously. If the committee had intended to appoint the assistant matron as matron why, asked the speakers, was that step not taken three months earlier? A strong opinion was expressed that the proceedings in regard to this appointment were not calculated to raise the reputation of the hospital in the public estimation. We were led to understand when the vacancy occurred that the appointment would be advertised, and we think it is very undesirable in the interests of Miss Shackleford and indeed unfair to her, that the appointment was not advertised when the vacancy occurred. The past management of this hospital has not been wholly satisfactory, and we hope more business-like methods may characterise the proceedings of its committee in the future.

### THE DISTRICT NURSE AND THE DUMMY TEA

THE annual meeting of the Wrockwardine Nursing Association was rendered exceptionally interesting by an address from Dr. E. Cureton. In the course of his remarks he said that district nursing is a branch of nursing of the highest importance, and requires the highest qualifications, because the district nurse has not, like the hospital nurse, a medical and surgical staff always at her call, and not many hospital appliances at hand. He proceeded to refer to the condemnation by a Colonial medical man of the dummy teat, which was defended by a nurse correspondent last week as "Baby's Comforter." The former, however, lays to its charge quite a number of the diseases of early life, such as adenoids and tonsillitis, deformities of jaw, with non-approximation of teeth, and consequently faulty digestion. The district nurse, Dr. Cureton affirms, can often do excellent work in enlightening mothers as to the proper hand-rearing of infants, and the very deleterious results of a more or less permanent nature which follows upon "the continual use of the dummy teat." We think that the knowledge is so important to the Empire that the training of district nurses should include instruction on the up-bringing of the young.

### PROBATIONERS UNDER A DISABILITY.

THE question of teaching probationers at Brighton Workhouse came before the Guardians at their last meeting, when the Rev. A. R. C. Cocks proposed the appointment of a resident medical officer, urging as the principal reason in favour of his motion that the Local Government Board will not otherwise recognise the probationers trained at the workhouse as eligible for the office of superintendent nurse. A long and acrimonious discussion followed, in which several matters not at all germane to the point at issue were introduced; and in the end the motion was rejected by 17 votes to 13. At present, therefore, probationers in the employ of the Brighton Guardians will continue to be under a disability which can hardly fail to affect their future career. The same disability also prevails at the East Preston Workhouse, and the Local Government Board have just formally intimated to the East Preston Guardians that any training given in their workhouse cannot be counted towards the three years' training requisite to qualify for the office of superintendent nurse. An East Preston Guardian, referring to the question at the last meeting, said that "the fact of the probationers being trained at their workhouse would not hurt their ultimate chances of becoming superintendent nurses with further training," oblivious apparently of the fact that probationers on finishing their course at East Preston are thus being condemned to work for three more years before they can qualify.

### CHRISTIAN SCIENCE AND NURSING.

We regret to see that trained nurses have again been concerned in a Christian Science case. In the Central Criminal Court last week a father was accused of the manslaughter of his son. The boy, who was ten years old, was sent home from a day-school unwell, and as he did not improve Frances Maude Turner was called in as a "Christian Science practitioner." Previously Miss Turner had been trained at the Middlesex Hospital for four years as a nurse. She stated that she believed the deceased was suffering from mumps, and prayed over him daily for several days. For this she was paid 4s. a visit, or £1 1s. a week. She was in no way dependent upon the practice of Christian Science from a monetary point of view, and frequently attended people for nothing. No one had appointed her as a practitioner; she had appointed herself. She admitted that she would take no more precautions in dealing with an infectious case than with any others. It is fair to her to mention that later on, as the boy got no better, she asked if the family would like to engage another Christian Science practitioner, or to consult a medical man. Miss Edith Jones, who is described as a "Christian Science nurse," confirmed the opinion of Miss Turner that the patient was suffering from mumps, but he ultimately died of diphtheria. Dr. Birdwood, medical superintendent of the Park Hospital, Hither Green, stated in court that he believed that the nurses were perfectly honest when they expressed the conviction that the child was recovering, and in the result the accused was found "Not guilty" of manslaughter, but "guilty of the misdemeanour of neglecting the child in a manner likely to cause him unnecessary suffering or injury to his health." He was ultimately discharged, but Mr. Justice Grantham signified a hope that "this would be a warning to him and to others that they must not neglect their children or give way to their own particular views." We wish we could think that the warning would have the effect of causing all trained nurses to give the Christian Science movement a wide berth. In the pursuit of their calling they often have the opportunity of mitigating pain or averting fatal results, but when they become Christian Scientists it seems that they cease to have the power to do either.

### £2,000 CLAIMED FOR "NURSING SERVICES."

The Lancashire papers have published under such headings as "Blackpool Nurse's Case," "Nurse and Patient: a Nurse's Romance," long accounts of an action brought by a married woman not living with her husband against the estate of a man who with his family originally boarded with her in her lodging-house. The evidence was to the effect that eventually she gave up the lodging-house and went to reside with the deceased, passing as his wife, and acting as his nurse while he was suffering for several years from cerebral degeneracy, which caused his death. The amount claimed was £3,000, and of this £2,000 was said to be due for "nursing services." The case was ultimately settled by the executors agreeing to pay £800 and a stipulated sum for costs. In spite of the newspaper headings, there was not a word uttered during the proceedings in court to indicate that the so-called nurse had received training of any sort.

### RESIGNATION OF THE MATRON OF GRANTHAM HOSPITAL.

We understand that Miss Sawle has resigned the post of matron of the Grantham Hospital in order to undertake that of matron of the General Infirmary, Hertford. It will be recollected that Miss Sawle, who was trained at Cardiff Infirmary and has had a useful and varied experience, distinguished herself on the occasion of the recent terrible railway accident. Thanks to her promptitude and foresight, the hospital was enabled to do such excellent work that lives were saved and immediate comfort and attention were given to all the injured who were taken to the institution. Miss Sawle's services on that occasion have received the grateful acknowledgment of the Great Northern Railway Company.

### ASSOCIATION OF TRAINED NURSES.

On Friday afternoon last week an address on the aims and objects of the Royal National Pension Fund for Nurses was given by the Secretary, Mr. Louis H. M. Dick, at the Hyde Park Association of Trained Nurses, 18 Cambridge Street, W., with the courteous permission of the principals, Sisters Wilcox and Reynolds. During the discussion which followed a nurse expressed her satisfaction at having the principles of the Fund authoritatively explained, and announced her intention of becoming a member—a course which she had been prevented hitherto from following owing to the extraordinary statements which she had heard respecting the Fund, and which she now realised were unfounded in fact.

### SHORT ITEMS.

The number of candidates at the last examination of the Central Midwives Board who were trained at Birmingham Infirmary and passed their examination was five, and not three, as stated last week.—The names of the three successful candidates trained by the Cardiff Queen's Nurses' Institute who passed the recent examination of the Central Midwives Board are Miss M. A. Jones, Miss Jane Graham, and Miss Frances Heddon.—The Countess of Aberdeen was present at the inaugural meeting, on Monday, of the winter session of the Irish Nurses' Association.

### The Nursing Outlook.

"From magnanimity, all fears above;
From nobler recompense, above applause,
Which owes to man's short outlook all its charm."

### NURSING HISTORY.

Mrs. Sarah A. Tooley has published through Messrs. Bousfield and Company, Limited, a book entitled "The History of Nursing in the British Empire." It contains an outline, of nursing before 1840, of the pioneer work of Elizabeth Fry, with an excellent portrait by George Richmond, R.A., and of the institution of nursing sisters. Mrs. Tooley then deals with Charles Dickens and nursing reform, gives a history of St. John's House, and the agreement entered into with King's College Hospital, with the events which followed. An account is next given of the Nightingale Fund Training School, the national subscription known as the Nightingale Fund, including an account of Mrs. Wardroper, of the iron discipline which was enforced at St. Thomas's School, and the work of Miss Crosland, Mrs. Wardroper's assistant, who created the office of home sister. All these chapters form interesting reading, but we miss the illuminating touch which could alone be given by a writer who has had the advantage of a personal knowledge of the pioneers of the nursing world. Mrs. Tooley is fully alive to the disadvantages under which she has laboured, for she states in the preface that she is "simply a scribe, with no claim to belong to the profession of nursing." This absence of knowledge on the part of the authoress probably accounts for the inclusion in the book of some photographs of persons who have little or no connection with the history of nursing. What justification can there be, for instance, for inserting the portrait of an official of an institution which has not been established more than ten years?

Mrs. Tooley is quite frank in stating that her "endeavour has been to take a brief for the history of the movement, unbiassed by any school or faction, and chiefly intent on following the main stream of facts and incidents." In consequence she has had little choice but to summarise the facts so far as she found them available. Her method of dealing with the more recent events in the nursing world appears to have been, to obtain her information from those who were willing to supply it, and to weave it in with a summary of certain facts as they have been published in our columns and elsewhere. It is not therefore surprising to find that there is a want of balance in the book which is manifested by the treatment accorded to various developments. The space and treatment extended to several of the most important bear little or no relation to the influence they have had in shaping the course of nursing in this country, and elsewhere. In dealing with hospital nursing and training schools Mrs. Tooley has almost necessarily failed to do full justice to the relative claims and importance of individuals and institutions. Where much information and photographs have been forthcoming much importance appears to have been given to the particular school, but no attempt is made throughout the book to estimate the position which each, from its work, is entitled to occupy on the roll of fame. Of course there is a great deal about Miss Nightingale, but no history of nursing can endure which does not bring out by evidence the extraordinary and world-wide influence exercised by Miss Nightingale in every hospital and institution in regard to the nursing and care of the sick. Every student of the history of the social development of nations, during the last sixty years, must find in official records, abundant evidence, that, wherever provision has been made for the treatment of the sick, there Miss Nightingale's influence has penetrated with results momentous for good to all concerned. The truth is, that, we can have no history of nursing in any proper or complete sense, until such a work is undertaken by someone, who has an intimate technical and general knowledge of all the developments which have taken place, and who has been in a position to know and appreciate how each change was brought about, what were the effects of these changes, and to whose influence and insight they may justly be attributed.

The book has chapters on various branches of nursing at home, in India and the colonies. They are, as Mrs. Tooley explains in her preface, "mainly summaries" of a neutral complexion, which indicate certain facts but in no sense cover the whole ground, or constitute a complete history of the divisions to which they relate. Mrs. Tooley has done her best with the materials she had available, but her book leaves a want unsupplied, for a history of nursing has yet to be written which is scientific in method, and will not only gratify the reader's curiosity about the past, but modify his view of the present and his forecast of the future. As a file of facts, more or less accurate, though by no means exhaustive, Mrs. Tooley's book may prove useful. No one can be said to write history, however, who merely supplies 400 pages of matter arranged in chapters with headings which include most parts of the empire, and entitles them "The History of Nursing in the British Empire." The illustrations, typography, and general appearance of the book reflect credit upon the publishers and printers. We have no doubt it will prove of interest to many who have no technical knowledge of nursing or its history.

### Nursing in Tropical Climates.

By ANDREW DUNCAN, M.D., B.S., M.R.C.P., F.R.C.S., Fellow King's College, Lecturer on Tropical Medicine at the London School of Tropical Medicine, and the Westminster Hospital.

### I.—THE NURSE.

(*Continued from page 99.*)

(2) *Food.*—Everyone knows that the greatest care must be taken as regards stimulants. Tonic water is an excellent beverage, whilst the efficacy of tea and coffee in the Tropics is manifest when we consider that the sun's action diminishes the action of the skin, lessens nervous activity, causes less carbonic dioxide to be exhaled, and induces cardiac paralysis. Now the above beverages have exactly the opposite effects. If stimulants be taken, the best form is whisky. Every nurse should possess a filter, and the investigation into the relative efficacy of filters some few years back showed that only the Berkefeld and Pasteur filters were of real use. Of these the Pasteur will not permit the passage of the enteric bacillus, whereas the latter can pass through the candle of the Berkefeld in from four to eleven days, so that the latter should be, if used, sterilised every three days.

*How to clean a Berkefeld Filter.*—It is well to know how to clean one of these instruments. The result of boiling a filter charged with bacteria is that the albuminous matter composing them become coagulated in the pores, and hence the filter is less porous, and after many boilings it may become impervious to water. There are various ways of cleaning the instrument.

(*a*) The best way is to dry it carefully in a water oven, and then burn the organic matter away in a muffle. This, however, cannot be done in an ordinary house naturally.

(*b*) Another method is to have two sets of bougies, one being used whilst the other is being cleaned. The latter is disconnected from the apparatus, all the metal parts removed, and the bougie immersed in 5 per cent. hydrochloric acid for a day or two: next it is boiled, but this is unnecessary if the first gallon of water filtered through the bougies after removal from the hydrochloric acid be thrown away. In this method the bacteria in the bougie are killed, and the albumin converted into acid albumin, which does not coagulate in the spores on boiling.

(*c*) Where only one bougie is in one's possession a very simple method is the following, according to the Berkefeld Filter Co.—namely, to place the bougie, under a tap, and gently brush it with a piece of loofah, or a soft brush. No soap or other greasy material should come into contact with the bougie. Lastly, in the process of purification of the drinking water, where you both filter and boil, always remember to filter first and then boil, *not* boil first and then filter.

With respect to solid food, Surgeon-General Maclean, a former Professor of Military Medicine at Netley, always enjoined that temperance was of great importance both as regards solids and liquids. He laid down that as a rule only one meal a day should contain meat.

(3) *Exercise.*—It is essential that every nurse should take some form of exercise during the 24 hours, thus preventing congestion of the various internal organs, especially the liver, that follow a sedentary life. Where compatible with her duties, the best time in the day is the early morning, after "chota haziri." Many take horse exercise where circumstances permit of this, and this is certainly the best and most exhilarating form. Should the early morning not be available, the exercise should be taken in the evening. One should never go out in the middle of the day. After exertion the nurse must take care never to get chilled.

(4) *Precautions at Night.*—The chief precaution to take is to avoid being bitten by mosquitoes, which have now been proved to be so hurtful in the propagation of disease. This is carried out by the use either of mosquito curtains, and here it is necessary to urge that these should be tucked in under the mattress and not hang on to the floor weighted at the bottom; or by punkahs, the flounce of which should have its lower border about one or two feet only off the face. When sleeping under a punkah the cholera belt must never be left off. Should the heat be very intense, much comfort can be obtained by sleeping on some matting.

(5) *Other Points.*—It would be well to take out a waterproof sheet, as these are expensive in the Tropics; a hold-all for one's bedding, such as can be obtained at the stores; a pillow and a good blanket. Lastly, the nurse should be cautioned never to bathe in cold water, but always in warm. I have only known one officer during my 20 years' service that was able to stick to his cold bath. My own experience is doubtless similar to that of many. After some months in the country I found I had a most troublesome pain in my right side, and on taking medical advice was at once asked if I took cold baths. On replying in the affirmative I was advised to take warm, when the pain, doubtless due to congestion of the liver, at once disappeared.

### The Nurses' Clinic.

### FRACTURES OF THE TIBIA.

THE after-care of fractures is not considered by most nurses to be one of the most interesting branches of their profession; and it is true that there is, as a rule, a certain amount of sameness about it. I have lately had the good fortune to work for a house surgeon who, being a bit of a genius, was allowed to do pretty much as he pleased; and as some of his ideas may be new I am venturing to give a short account of a few of his cases. Several were those exceedingly awkward oblique fractures of the tibia, very near the ankle, just where the pull of the muscles makes it most difficult to retain the fragments in position.

The patients were all men about or over fifty years old, and all alcoholics. The first case, which came in before the advent of our new house surgeon, was put up in the usual way, with a long back splint with a foot piece and two side splints, thus fixing the joint above and below the fracture. No massage was ordered till four days after admission, by which time the leg was much swollen and sodden. The fragments were never even approximately in a good position. The massage was continued for twelve days, by which time the swelling had gone and the general condition of the leg was much better, but the fragments were in no better position. Plaster was tried without success, and finally the bone had to be screwed.

The next case was at first put up on a Cline's splint, and the position of the fragments was as bad as in the first case; then our new house surgeon took things over, and on the third day it was put up in the following manner :—

First the tendo achillis was divided through a small incision; the wound was dressed with gauze and a thin bandage applied. Over this a small stirrup extension was applied coming only to the ankle joint, and made to wrap well over the instep. The leg was then enveloped in a sheet of Gooch splinting, reaching from just below the knee to the bottom of the foot and having a piece cut out for the heel. This was secured with two bits of webbing and buckles and a weight of about nine pounds attached to the stirrup, which, of course, ran on a pulley over the bottom of the bed. This case, like all those that follow, was treated with massage from admission, and they also had passive movements after the first week. The recoveries were excellent, with no deformity or stiffness.

The third case treated on the same principle was that of a cab-driver, who came in with a compound comminuted fracture of the tibia. This fracture was higher up the leg, just below the middle of the tibia. There was a great deal of cramp and muscular spasm in the thigh. The patient said he always suffered from it. This spasm was partially relieved by slinging the leg with the knee flexed and by massage to the thigh, but in spite of everything it kept returning for about ten days. Union was delayed for about six weeks, which was not astonishing, considering that the bone was broken and splintered into a great many pieces ; but the final result was very good.

Another interesting case was that of an old man, who said he did not know how old he was, but he was sure he was over seventy-five ! He looked quite eighty and was very frail. The injury was a very oblique fracture of the femur, extending upwards from close to the knee joint.

This leg was laid on a MacIntyre splint and very lightly bandaged to it, with Gooch splinting in addition round the thigh. The house surgeon could not use his favourite Thomas' knee splint, as the man was so old and his skin would not bear the slightest pressure. It was taken down and massaged daily and the knee moved about once a week.

The patient was very weak and developed pneumonia, and in spite of all our efforts to keep him propped up in bed he used to slip down three or four times a day. In fact, so little rest did the leg get that I expressed my doubts of the bone uniting, and in return was told that the only bones which never united were those in which no movement between the parts was possible—*i.e.*, the bones of the skull.

It is certain that in six weeks there was good bony union, and the splint was removed, more vigorous massage and passive movements were given, and in another month he was able to get about on crutches and to stand alone and had good movement of all his joints.

### A Nurse's Province.

The real limits of a nurse's province, what it is and what it is not, is sometimes a rather difficult point to decide. It is more often from ignorance than from wilfulness that a nurse takes a long stride and finds, considerably to her own surprise, that she is straying into the province of the doctor. I do not think that anyone who knows anything about the examinations which a nurse has to pass before getting her certificate will deny that a trained nurse must be an intelligent woman. Therefore it is not to be wondered at if, after several years of experience, she is able to do many things which are in reality the work of the doctor. Has she not stood by his side and assisted him dozens of times? She may not have read the text-books on certain operations, but she has observed, and so knows from experience, the various stages and what is needed. Can it be deemed vanity in a nurse if, in her own heart, she feels as she looks at a student or a young house-surgeon, performing his first operation, with none too steady a hand, that she could have done as well, or even better? Not that he did not know his work; but because she had stood by while the same thing had been done so many times till the needful routine had burnt itself into her memory. In an amputation of the finger, for example, the young man in his nervousness, perhaps, has quite forgotten that the tourniquet should be removed before he stitches up. The nurse hesitates ere she hands the stitches, expecting to be requested first to remove the tourniquet. When asked in a very abrupt manner "to be sharp with the stitches," she feels that at this point she would like to ask whether he did not wish the tourniquet removed. But fearful lest she should stray beyond her proper province, she quietly does as she is requested when a remark from one of the surgeons that "it might be as well to remove the tourniquet before sewing up" shows the young operator why it was that the nurse did not promptly hand the stitches. That remark might have been saved had not the nurse felt that she would be exceeding her duty to make the inquiry.

A nurse may know and feel all this, but she must remember that she is still only a nurse. Of course, experience will make her of great value to the doctors she may work under. But she must never let it be said of her that she has overstepped the boundary line by prescribing a remedy for ever such a simple disease, or by giving an opinion upon any matter which belongs to the doctor's province.

But, alas ! there are nurses with whom one meets as one goes through the world who have no hesitation in taking upon themselves the diagnosis of a disease and as readily prescribing a remedy for the same. They talk of their cases and successes as another girl speaks of her triumphs at tennis or golf. What is more unprofessional than to hear a nurse say, "The doctor thinks the patient is suffering from such and such a disease, but I'm sure he is mistaken; I think it is so and so"?

Yes, she may think. That is the right of all men and women, and I should have but a poor opinion of any nurse who had a case of observation under her care who did not consider the various symptoms and in her own mind form an opinion as to the disease from which the patient was suffering, but there the matter should end. There are times when a nurse is called upon by the doctor to do certain things which do not properly belong to her province, but it should never lead her to attempt the same things on her own responsibility. When she acts at the command of the doctor, the responsibility is his. A nurse who in all ways studies to become an intelligent helper to the doctor, a woman whom he can feel instinctively has the interest of his case at heart, can hold up her head and look the whole professional world in the face, and fear not the criticism of any man, for she walks fearlessly in her own domain.

### Incidents of the Christmas Season.

WRITTEN BY NURSES.

The feature of our special Christmas number this year is a series of incidents occurring at the Christmas season contributed by nurses in response to our invitation twelve months ago. For a considerable time past we have published under the heading of "Incidents in a Nurse's Life," more or less interesting particulars of the experiences of nurses belonging to all branches of the profession. Some, of course, are not so informing as others, but they all have the advantage of being personal narratives, and as a rule they illustrate the drawbacks or the difficulties which the average nurse has to encounter in the pursuance of her calling. The other side of the picture, the sense of satisfaction due either to the performance of useful work, or to pleasure derived in affording help to others, is fitly brought out in connection with the rejoicings of Christmas. Our contributors, it will be seen, relate incidents which happened in various parts of the world, and under widely differing circumstances. Although the opening of the New Year is the great Festival in Japan, an English nurse was able to introduce Santa Claus in Yokohama for the benefit of her patient's child. A member of the Indian Nursing Service tells how Christmas was spent in a military hospital; an Army sister shows how she forgot her hatred of the heat and of night duty at Christmastide in South Africa in doing a kindness to the black dependents among whom her lot had fallen; and the romantic element finds a place in the contribution of an English nurse who spent her Christmas Day among Californian gipsies. There is a contrast between the account of the observance of the festival in distant Newfoundland and a well-known London hospital, but not in the spirit which dominated it; while a nurse in another great metropolitan institution links in the Christmas season with an incident which testifies that confinement in the wards may mark the turning-point in a downward career. There is the right ring of merriment pervading the proceedings at a typical cottage hospital, and there is the true note of pathos in the visit of the father engaged on a night job to his foster child on Christmas Eve. This last is a note which can never be absent in the care of the sick. But the nurses who, whatever the sphere of their duties, do their utmost to turn the attention of their patients to the joyous aspects of the season, are fulfilling the lighter responsibilities of their avocation; and those who have recited their experiences may be congratulated upon the practical proof which they have given that wherever they may be located, or under whatever sky they may be working, there are no conditions in which nurses cannot invest the celebration of Christmas festivities with elements bringing happiness to others.

### In Japan.

Just a week to Christmas and on this particular day, being off duty and the outside world not specially attractive, I settled myself comfortably for a long morning of letter writing for the home mail, hoping just a little that my services would not be required till the letters were finished. Alack! before I was well started, in bustled our old house amah announcing that the telephone required my presence. After a little delay the telephone boy switched me on to the Yokohama connection and I had orders to come down as quickly as possible—"Urgent case, no time to be lost."

With all speed the last items were thrust into my trunk, my outer garments were donned, and I was making quick tracks for Tokyo Station in a jinrickisha, while my luggage followed on another. Only three minutes to spare before the train departed, but an active little red-capped porter seized my big trunk, while the jinrickisha coolie followed with the bag, and with their aid—most graciously given—I managed to struggle through.

A driving rain completely blotted out the country through which the line runs, so I amused myself by watching my travelling companions—an autocratic old gentleman clad in a kimono, an Inverness cloak, and a "bowler" hat of ancient pattern, bright yellow kid gloves, and a sort of fur boa; a pretty little Japanese girl of about eighteen, with very much dressed hair and a gorgeously smart silk kimono partially hidden under her rain kimono; and two rather aged and wrinkled ladies who smiled and talked a great deal. The old gentleman had provided himself with two air-cushions and several rugs. Each of the elderly dames proceeded to inflate the cushions while the little girl arranged the rugs, but it was quite ten minutes before his lordship settled down. Then the youthful damsel fished around in a capacious silk travelling bag and unearthed refreshment in the shape of "ame" (a sort of Turkish delight), which she cut into small cubes and handed round on wooden toothpicks. After that came a great deal of dry hand washing with Japanese paper handkerchiefs.

By the time we reached Yokohama the rain had ceased and the sun was struggling through. This was a great relief with a jinricksha ride in view—it is so intensely uncomfortable to be packed up with a hood and apron in one of these adult perambulators.

On arrival at the address given the house-boy greeted me with the information that his mistress was "not at home," whereat I felt a trifle discomfited. His English and my Japanese were both deficient, and the situation was becoming somewhat trying, as he steadfastly refused to admit me, when a house amah with a goodly knowledge of English came to the rescue and ushered me upstairs with many apologies.

My patient proved to be an American—frail, highly strung, and nervous to the last degree. "I am so glad you could come, nurse; but I don't know where you can keep your clothes, and I am afraid you will have to sleep in my room, and I have all my Christmas preparations to make, and the doctor says I must not see any visitors, nor write any letters, but keep perfectly still and quiet, and I *can't* do that."

Poor dear, she looked a most miserable little soul, especially as her bed had not been made for several days and her general appearance was more or less unkempt. I assured her that my trunk was accustomed to serving as wardrobe, bureau, and that a bed in her room would exactly suit my requirements, under the circumstances. The doctor had left a string of directions, which my patient detailed to me, while I removed my outer garments and scrambled into a cap and apron. Suddenly the door opened and in rushed a quaint little figure, clad in pyjamas and a padded kimono, with long curly hair tied on the top of her

**IN JAPAN**—*continued.*

head in a tight knot. At the sight of me she stopped short, demanding, "Why has this doctor woman come here, mother, and what does she wear that handkerchief tied on her head for?"

Her mother introduced her to me as Paddy, and explained that when she (the mother) was sick in Los Angelos

A Japanese Nurse.

the previous year, they had had an American nurse to whom Paddy greatly objected. The mother explained to the wee girlie that I had come to try and make her better, but at first Paddy regarded me with dislike and distrust, though we very soon became fast friends. Paddy's amah appeared on the scene to carry off that young person for her midday siesta, and I set to work. The first few days were anxious ones, but by the morning of Christmas Eve my patient was so much better that we ventured to sound the doctor (a jovial old Irishman) on the subject of Christmas festivities. Paddy had been promised a Christmas tree (or rather she had been told that Santa Claus might possibly leave one for her if she was very good), the gardener had fixed the tree on an elaborate stand, and lots of toys, candles, etc., had been purchased. At first the doctor shook his head and told us to wait until New Year at any rate, but that, of course, upset the Santa Claus theory, so he finally gave a grudging consent to the tree being fixed up in my patient's room, on condition that she did not sit up in bed, but would be content to lay and look on. I spent my off-duty time rushing round in a jinrickisha, purchasing tinsel, spangled gauze, gifts for the native servants to hang on the tree, and the like, while Paddy's father was presented with a list of many articles for the festive season, including holly, flowers, candy, mince pies, etc. We beguiled Paddy into retiring to bed half an hour earlier, also coaxing the old cook to let us have an early and "quick time" dinner, and then we set to work. It took four able-bodied men to convey that tree upstairs, for it stood over six feet without the stand. The gardener had done his very best and the stand really was very fine, representing a Japanese compound, with a typical garden, a pond with a boat, etc. Two friends had arrived to help, and we worked like niggers till nearly 11 o'clock. The tree looked charming, but the room was littered from end to end with wrappings, empty boxes, discarded toys, odds and ends of all sorts; my bed having been used as a dumping ground for undesirables.

In the grey dawn of Christmas morn, before I was well awake, two little hands came dabbing on my face and a shrill voice piped in my ear: "Did you see Santa Claus come down the chimney, and did you tell him I was good?"

Whereupon I answered that if I had looked at Santa Claus he would assuredly have melted away.

New Year is the great Japanese festival, but the servants in foreign houses rise to the occasion at Christmas and from the No. 1 boy to the bath coolie we received greetings. While we were busy on the tree the boy had worked in the hall and downstair rooms, making everything look very gay. Japanese holly has no berries, but they have a red-berried plant rather like a small mountain ash and the natives fasten these berries among the holly leaves with very good effect. Mistletoe is plentiful, also palms, and with poinsettias, Christmas roses, bamboo and dwarf plum trees in blossom (a great feature at this season and New Year) we had a goodly show.

After breakfast came Christmas gifts—such an array of toys for Paddy, with ornaments and books for her parents, and I had not been forgotten, quite a number of pretty and useful things straying my way. Some of the presents were rather quaint—the laundress brought a big bag of cube sugar, the coolie's aged parent some weird sweetmeats, etc. About 11 A.M. the worthy doctor arrived with a broad smile and many cheerful greetings. My patient meekly asked for a little Christmas fare, so he said she might have a little turkey, but no pudding, mince pies, nor cranberry sauce—those could wait till the 4th of July. She begged for ice-cream, whereupon he smole a broad smile and responded: "Certainly, on condition that your cook puts it in a hot oven first."

I should have loved to go to the English church, but could not leave my patient, who was already beginning to show

An English Nurse.

signs of fatigue and excitement, so I spent my spare moments in arranging candy, bonbons, fruit and cakes, for our guests—the native servants—when they paid their visit of state in the afternoon. After tiffin we lighted up the tree and then our visitors began to arrive, clad in their holiday kimonos and armed with "furoshikis" (a sort of big Japanese handkerchief cloth used for tying up things in) for their gifts. They all made profound salaams to us in turn, beginning with the master of the house, then squatted in a ring round the tree, broadly smiling with delight at the various mechanical toys which were wound up for their edification. Then we handed round refreshments, all of which were carefully consigned to the furoshikis, and more salaams were made. After that their gifts were presented—more salaams—and finally they filed out, beaming with satisfaction. By this time my patient was fairly tired out and the room required a big airing, so we quickly removed the tree and its appurtenances to Paddy's nursery and once more peace reigned. We all had a very light and wholesome dinner and retired early.

Little Paddy's prayers that evening included thanksgivings for the visit of Santa Claus, also for her mother's recovery, and I think we all felt that we had special cause for gratitude that Christmas night.

### An Idyll of the East-end.

Mrs. Sam Brown, of Paradise Court, was the centre of attraction, of which Mrs. Dan'l Stubbs was profoundly jealous, even though the cause of the attraction was an accident to Mrs. Sam's husband, who had been taken to the hospital.

Paradise Court needs little description; it was of the very usual type of the East End—dirty and uninviting to the last degree, and the usual interior of the houses was like unto the exterior. With the support of the doorpost Mrs. Brown maintained a fairly upright posture, and from this vantage-point she was giving in detail, with embellishments thrown in, the account of her "old man's" accident and his final reception into hospital.

"Yus, me 'eart was in me mouth when the police come with the news, and what with the thought of 'aving to bring up all the children, and what with the thinkin' of him dead, I was fairly off me 'ead. The police, he says, ''Ere, missus, don't take on so, 'e's only bin and broke 'is leg and cut 'is 'ead open and 'urt 'is 'and, they'll soon patch 'im up in the 'orspital,' so I dries me eyes and fetches me shawl and I goes along double quick to the 'orspital." Here she changed her position to the other door post and paused for breath, and, having filled her capacious lungs with a fresh supply, she proceeded to tell the still eager and sympathetic audience of the interview at the hospital.

"When I gets there the porter 'e says ''Ere, what's yer want?' 'Why, me 'usband,' says I. 'Well, oo's 'e?' he says. 'A man with a broken leg,' says I. 'That's where you'll find 'im,' he says. So I gets to the haccident ward and I goes in and was just agoing to see 'im when a young woman comes to me and says a bit uppish like, 'Would you mind waiting at the end of the ward a minute. Which patient do you want to see?' 'Why, me 'usband, o' course, an' 'oo's a goin' to stop me?' says I. 'If you would tell me your 'usband's name I'll take you to 'im,' says she; but the doctors was all round 'is bed, so I 'ad to wait."

"When I did get to 'im I says 'Well, Sam, 'ow'd yer do this 'ere?" Sam explained in his own formula that the kerb was all to blame and that they should'n't have such high pavements when one has to walk home late at night. Incidentally he mentioned that they had been having "a drop o' beer at the Angel" and that Dan'l Stubbs wasn't the best behaved chap at times, and that his fists were rather hard. And certainly Sam's black eye gave emphasis to that statement.

"When I 'eard that 'e 'ad 'ad some beer I just goes for 'im, an' I says, ''Ow's the rent to be paid now, an' yer children fed?' 'E didn't speak, and 'e looked that shamefaced. I never 'eard 'im so quiet-like afore, an' I 'opes it'll be a lesson to 'im to treat 'is poor wife better."

So, with many comments on Sam Brown's conduct, and many avowals of being Mrs. Sam's best friends, the party dispersed to either do or neglect—probably the latter—their household duties.

With Sunday came visiting day and visiting day brought Mrs. Sam Brown to the hospital once more.

She still thought him "quiet-like and shamefaced," but had she really known it was Sam's conscience doing its best for him, and he was beginning to repent of his neglect and selfishness. Thoroughly sobered down and with plenty of time for reflection and review, he realised that exactly ten years ago he had married Sally, who was then a smart young woman. Yes, he remembered how proud he felt when Sally appeared in her bridal garb—the petunia velvet frock, flashing with bright sequins, and the wonderful large hat, with the still more wonderful large feather surmounting a mass of woolly, becurled hair arranged over the ears in the special fashion of her class. To think of it! Here she was to-day in a ragged old black skirt, a bit of a shawl, and a headgear unrecognisable apart from its owner's head. "Yes, Sam," he thought to himself, "it's all your fault." Suddenly a vision of the identical petunia colour floated by, occasioned by the arrival of the new wife of No. 21, whose bed was opposite Sam's bed.

Mrs. Sam gazed at her with envious eyes, and her husband followed and read the meaning of her gaze.

"'Ere, I say, Sal, 'ow would yer like a git-up like that?"

"Ga on, fine and likely, and you in bed 'ere not earnin' a 'apenny." "We'll see one day, Sal my gal," he said with more affection than he had been accustomed to use lately. Somehow tears came into her eyes at the tone of his voice, and she made her mind up then to forgive and forget his past misdemeanours.

"To-morrow's Christmas Day, Sal, got anythink for the kids?" "Made 'em a pudden, that's all, can't expect anythink else, and their father bad in 'orspital and all." "Ah." Sam rejoined, "another Christmas and we'll 'ave the beef as well as the pudden." Here, again, Sal felt moist about the eyes and surreptitiously brushed away a troublesome tear with the poor, work-begrimed hand.

. . . . . . .

Christmas morning dawned on home and hospital alike. Although there may have been many feelings of home-sickness afloat yet the patients were almost hilarious in their good spirits. All through the day the brightness and cheeriness was maintained, and at night, when "lights-out" time came, though two hours later than usual, the patients declared that the day had been all too short and everybody too kind. Sam was loud in his praise of everything, and declared that "'E 'ad never 'ad sich a rattlin' good time afore, and all without beer!" "Look 'ere, lads," he ejaculated to his companions, "I'll tell yer what; I'm goin' to chuck the drink after this, so good-night," and he slid down amongst the bed-clothes—partly to be out of line of fire, some of which was ridicule, whilst the majority was genuine approval, and partly to hide an unmanly expression emphasised by suspicious moisture of the eyes.

. . . . . . .

There was another meeting in Paradise Court. "Sam

AN IDYLL OF THE EAST-END.—*continued.*

Brown's missus" was the only absentee, for the Brown family no longer lived in this *élite* quarter. They were, however, the topic of the hour and the direct cause for the gathering of the inhabitants of the court.

Mrs. Daniel Stubbs's enviable spirit once more asserted itself, and again she coveted Mrs. Sam's good luck and notoriety.

"Only yisterday I see 'er a walkin' along like the Queen 'erself," she was saying, "and 'er little gal in red and 'er old man with a white collar and a beautiful green tie and all on."

"Goin' to church was they?" jeered one.

"You jest 'old yer tongue, there, Mrs. 'Awkins; do you an' your old man good if you was to go, anyway," shouted an ally, and Mrs. Stubbs brought the meeting to a close by remarking, "Well, well, some folks 'as all the luck, it was all along o' that there haccident and the good they done 'im at the 'orspital."

### In South Africa.

Pouf!—how hot it was! I turned round again and endeavoured to find a cool spot in bed, but failed. The mattress seemed burning. Then I tried sitting up with a pillow behind me; but it was worse than before. Was there ever such a suffocatingly hot thing as a feather pillow at one's back with the thermometer at about 120°? I got up and sponged my face and hands, straightened the hot, tumbled sheets, and again lay down in the hope of getting even half an hour's more sleep. It was about 11 A.M., and I, who was on night duty, had had about an hour and a half's sleep; and I knew that, once well awake, sleep in the heat of the day was, with me, impossible. About six o'clock, when dark, or nearly so, I might drop off again; but we dined at eight o'clock, so I could not hope to secure much rest at that time. I tried to fortify

A Bridal Group in the Transvaal at Christmas.

myself with the saying of a physician, "It is not the want of sleep that matters, it's the worrying about it." "I won't worry," I said fiercely to myself; "I'll keep perfectly still and—oh, bother! get out!" This last remark was addressed to a pertinacious mosquito, who, having invaded my mosquito-net, had to be caught and killed before I could put my resolution into action. I composed myself once more with an aching head, feeling a trifle more miserable than before. I hated the heat, I hated night duty, and I felt just then that I hated even Christmastide. It had been settled by the authorities for excellent reasons that Christmas was not to be officially recognised, and as I fretted my mind drifted back to the Christmas before, when everything had been so different.

I was then night sister in one of the big London hospitals, and as I made my rounds I thought each ward more beautifully decorated than the last. Truly they were like fairyland, with their delicate festoons of greenery draped from the ceiling to the walls, and Chinese lanterns and dainty lamps peeping from among the leaves. Sister's room was piled high with toys for the children and useful gifts for the elders. Such quantities of dolls, books, horses, carts, jacks-in-the-box, drums, soldiers, bricks, scrap-books—it was a veritable toy-shop. Then there were handkerchiefs, underclothing, shawls, woollies, petticoats, knick-knacks, and other gifts too numerous to tell, all marked off for the happy recipients.

By the time that I had in imagination distributed all the gifts, given my good wishes to the patients, and witnessed their gratitude, I had worked myself into quite a good frame of mind. I recalled how many old friends had come to revisit their Alma Mater on that Christmas Day; how we had talked over old times and gone together to see our old wards, where we had spent such happy times; how we went and saw a dear old granny who, though now, alas! on her deathbed, was still cheerful and patient, and how she had pointed proudly to the warm, soft shawl she had received; and as we teased her about the pretty colour matching her blue eyes she had answered back as merrily as ever. Her youngest grandchild, aged one month, had been brought to see her, and she whispered that it was to be his, "arter I done with it!"

At this juncture my door was softly opened by Sister S——, who murmured: "I didn't want to disturb you, but I fancied you would be awake and might like tc have your letters; the mail is just in, and there are two parcels as well." Could any mail have been more opportune? Here were Christmas letters and cards, and even a grand plum-pudding in a much battered tin! "And I have got some chocolates and figs for dessert, and received a chicken," chimed in sister; "so we shan't do badly after all. But oh! Sister, I must tell you, Whiskey (our Kaffir servant) went to get married yesterday, you know, and he wants you to photograph his bride and himself. I told them to come up here, and if you were awake—why, here they are; quite a party!"

I jumped up, threw on a cotton wrapper, tucked up my hair, and looked out. There was the bridal group, radiant with happiness and good temper, and decked out in the gayest colours imaginable, grouping themselves for the photograph. It was impossible to refuse them, and I am glad to say that the picture gave great satisfaction; and whenever I look at it I recall that Christmas Day in South Africa, which, after all my dismal prognostications, was in reality far from being a miserable one.

### In a London Hospital.

Christmas-day fun started early in "Grosvenor," the children's ward of the London Temperance Hospital. At six o'clock the busy night staff nurse dispensed breakfast to her large family, and the toys and stockings placed at the side of the cots by the fairy god-mother the night before were pounced upon by eager hands, and shouts and squeaks of delight were heard on all sides from little beings, who till now had not known the meaning of Christmas and the joys it brings in its train.

We day nurses came on duty in the wards at the usual time, 8 a.m., after flying visits paid to one another's bedrooms, and breakfast at which home sister did not mark anybody "late." The day was begun with carols, and sister and the four "Grosvenor" nurses, with a convalescent baby (well on to recovery) tucked away under an arm, proceeded to "Sturge," the men's surgical ward, which is on the same flat, on singing intent. Of course we led off with "Hark, the Herald Angels," amply backed up by the men, and a violin played by one of the "Grosvenor" nurses.

A unique sight was "Sturge" that morning, with its white glazed-brick walls contrasting well with the holly and ivy decorations, and the red nightshirts of the men, to whom we had bequeathed our small charges *pro tem.* The arrangement seemed quite satisfactory to big and little patients; the latter, sitting on the beds or cosily tucked away, staring wide-eyed at their new nurses. At the farther end of the ward was sister at the piano with her seven handmaidens, as she sometimes called us, grouped round, leading the carols with right goodwill; outside, the grey December sky and atmosphere, which only served to emphasise the warmth and cheeriness within.

But work there was in plenty to be done, and even a quarter of an hour out of the routine makes a difference, so we soon hurried back to our ward with the babies, to dust beds, polish "brights" and lockers, and get on with the dressings, which on a full surgical side demanded attention of sister and staff nurse for a good hour.

About ten o'clock matron came in, with a hand-shake for each of her nurses and a word for every small patient, and especially for any little one too ill to take more than faint wondering interest in the merrymaking around.

Twelve o'clock is the dinner-hour for patients in our hospital, and on this particular day the "Sturge" and "Grosvenor" nurses joined forces and all helped to distribute the turkey carved by the house surgeon in the men's ward, and the plum-pudding helped by sister.

About three o'clock music was started, and such of the men as were convalescent were allowed to smoke. We had many visitors, and it was somewhat comical to see our jolly junior resident packed into a miniature armchair with a knowing baby boy, "Georgie," of eighteen months perched on his knee. The baby wore a long red nightgown, and on its head a "mastoid" bandage, much resembling a night-cap. He laughed and crowed and thoroughly enjoyed being made to keep time with a diminutive arm to the nursery rhymes being sung; and when a summons to "Casualty" hurried the young doctor away there was a pathetic howl which only the privacy of the bath-room could quell.

In the middle of our tea in the ward kitchen a steady tramp as of "armed men" was heard, which is generally associated with a "stretcher case" for the surgical wards; but to-day it was only the porters with poles and stretchers come to carry down the first instalment of patients to "A" ward, where the Christmas-night entertainment is annually held. The children went down later, carried by sisters and nurses.

As it is quite a home affair, no outsiders were among the audience, which, as well as the patients, consisted only of nurses, maids, porters, and a few hospital friends. The performers were the nurses, house surgeons, and one or two sisters, and the patients enjoyed the applause that a nurse belonging to their own particular ward gained for song, solo, or clever piece of acting, she on her side feeling amply repaid for off-duty time given up to rehearsals by the hearty claps, or a "well done, nuss," as she left the platform. About eight o'clock, after a pleasant two hours, the programme came to an end. There was a general move, and porters once more appeared to carry helpless patients back to their wards, whilst doctors and nurses conveyed small people, casting longing backward glances at the big tree, to their spacious nursery once more.

After supper, lights were put out, and in an hour's time all was in silence once more as day nurses wended their way to welcome bed, and the night nurse started on the round of her sleeping charges to find one wakeful bigger child, an especial pet, who had tried hard—and successfully—to keep awake to tell "*her* nurse" all about the wonders of the day.

### In Newfoundland.

We had all hoped that we should have snow on the ground for Christmas. The weather a few days before had been mild, so that we had been rather fearful, remembering that "a green Christmas makes a fat churchyard." But Christmas came bright and clear and frosty, plenty of snow down, and just as Christmas ought to be. "Drift" later on in the day made us realise less pleasantly that it was Christmas weather indeed.

We began to decorate the wards several days before—paper flowers, mottoes, flags, the Union Jack and the Stars and Stripes side by side, together with some holly with its red berries, come all the way from England. Chinese lanterns shaded prettily the electric lights.

"Johnnie," our youngest patient in the men's surgical ward, had never heard of Santa Claus. His home was in a northern outpost which Santa Claus evidently had never visited, so he had to be told how to prepare for his coming by hanging up his stocking, that he might get some of the gifts brought for good children on Christmas Eve.

Johnnie was not always good, and promises of reward and threats of punishment were alike powerless to work a reform. "Did you do this at home, Johnnie? What did your father say?" "He used to jaw me and bate me." "If you do it again, I shall put you in the bathroom to sleep." "Yes," Johnnie said, very thoughtfully, "and I 'low I won't like that very well."

When Johnnie opened his eyes on Christmas morning great was his excitement. Santa Claus had given him the lion's share of his good things. "Dickie and Howard had things in their stockings, but I 'low I got the best," and they pulled Tom Smith's crackers for the first time in their lives.

The Christmas dinner, for those who could eat it, consisted of roast goose or turkey, vegetables, and the orthodox plum pudding, with fruit later on. Some of the older patients on the male side amused themselves trying to solve a picture puzzle, for which prizes were offered by one of the daily papers. The prizes were not very valuable, but one of them came to the hospital.

In the evening a magic lantern provided amusement for

### IN NEWFOUNDLAND—*continued.*

an hour or so. That, too, was quite new to some of them, living, as the greater number of our patients do, in small coves and bays round the island, where magic lanterns are

Silver Thaw in the Hospital Grounds, St. John's, Newfoundland.

unknown. But the great event of Christmas was to follow a few days later, when the ladies of the Cowan Mission gave a tea and concert in the men's surgical ward. All those who were well enough to be moved were brought over, and tea was served as well in some of the other wards, where the remaining patients were sufficiently numerous to make it worth while.

Each adult patient was given a Christmas card with his name on it and a dollar, the latter being a highly appreciated part of the entertainment. The nurses had a very dainty handkerchief, and even the matron and nursing superintendent were not forgotten, but were each presented with a book. The children had toys, and Johnnie came to the conclusion that he would "bide here" and not go home any more.

Soon after Christmas we had a "silver thaw." I fancy in England it is something of a novelty, but it is a not uncommon sight here! The rain comes down quickly and quietly to the cold earth. Where it falls it freezes, until every branch of the trees, every house, every telephone wire, is covered with a coating of ice, sometimes two or three inches thick. The trees bend their branches to the ground. The umbrella used as a protection from the rain will not close because of its icy covering. One's hair has tiny crystals hanging from every stray end. Electric cars keep on their way with difficulty because of the coating of "silver thaw" which forms on the wire overhead and on the rails below more quickly than it can be removed; and the city is illuminated with lightning-like electric flashes from the cars in their vain endeavours to keep going. It is a weird scene at times, but when the sun shines next morning what a beautiful picture it becomes. The glitter of the sun on every tiny ice-covered twig is almost blinding. One has to turn the eyes away from its dazzling splendour. But it is cruel, too, for scattered around everywhere are

Newfoundland Christmas Patients.

branches, large and small, of trees broken by its weight. It works destruction among the fruit trees of the gardens, and when it comes in spring, just as the buds are about to burst, its cold embrace crushes and kills them.

### In a Punjab Military Hospital.

To some people who have never been abroad, "India" conveys the idea of perpetual punkahs and iced drinks, and the very word "Christmas" seems out of place; but to those whose work takes them to Northern India the impression left by December and January is very different. Although we have, for the most part, intensely blue sky and bright sunshine, the cold icy wind which often blows straight off the snows crowning the Himalayas makes big wood fires pleasant even in the day, and plenty of blankets acceptable at night.

As nothing is done by Government to celebrate the festive season in the Army, everything possible is undertaken by the regimental officers in barracks, and by the nursing sisters in hospital, to make things less dull.

Last year we thought it better to spread our small festivities over three days, and to begin with beat up recruits for a smoking concert on the 23rd. We were very busy in consequence of a severe outbreak of pneumonia in one of the regiments, and also short-handed through the vacancy caused by the marriage of one of our sisters, so preparations were made under difficulties. However, willing helpers in the shape of orderlies off duty soon transformed one end of a ward in a block of minor cases into quite a charming drawing-room, with rugs, chairs, etc., from the sisters' quarters; made to look very Christmaslike by shields with all the old familiar greetings. Most of the latter had done service many times out here in various Punjab stations, and were the relics of a Christmas long ago spent in a London hospital.

By 5 p.m. on the day, all surgical patients allowed to be moved, and a few (alas! very few, as so many were in the acute stage) medical convalescents, had been brought over, and a crowd of men, not from the sisters' blocks, made up a large audience, and soon cigars and cigarettes were in full swing. In spite of Christmas shooting leave and the Pindi manœuvres, a great deal of local talent had been dug out, and a most popular subaltern brought down the house by his first song, and, in response to an encore, gave the old-time favourite, "Sally in our alley." One of the medical officers with a splendid tenor voice was called up again and again; whilst another made the men roar over his coster songs. The two little daughters of the civil surgeon did several step-dances so unconsciously and prettily that they received a great ovation, and when they finished up with the "hornpipe," in sailor frocks, the men clapped again and again. The small maidens were so impressed by their enthusiastic reception that the elder one afterwards announced her firm intention of turning into a nursing sister some day. As may be imagined, the ladies with voices had responded nobly, and by 7.45 a very tired but pleased audience were conveyed back to their respective wards.

Our next entertainment was a Christmas tea to our own patients on the 24th, and very charmingly the men had arranged the verandah in our surgical block—long tables with snowy cloths, decorated with flowers and large bunches of mistletoe (the latter from the Himalayas) made the scene correspond as nearly as possible to our home wards. The tables literally groaned under stacks of bread and butter, jam, and large pink and white sugar covered cakes, whilst bon-bons filled in the spaces. It is the one day in the year when Tommy Atkins has his tea made in a teapot, and is able to help himself to milk and sugar, and it is much appreciated if one may judge by the number of cups poured out. The orderlies on duty came over for their tea as the sister whose turn it was in the wards could spare them, and the senior sister's bearer, who has by this time had a good deal of experience in these matters, had to put by cake for the late-comers. One item in these feasts is always a plain cake for convalescent enterics, and oh! how hungry the latter are, so that one has to call out warningly that Privates Brown and Jones must not even look at the sultana ones. One year we were obliged to have a little special tea-room for these delicate convalescents alone, but this season pneumonia instead was rampant.

Christmas Day itself, of course, spelt a dinner of sorts, and we had prevailed on the Senior [illegible]ical Officer to allow all diets to be drawn as "roast beef," and some really respectable sirloins appeared in our surgical verandah. The tables looked very festive with piles of oranges, walnuts, and bon-bons, and the medical officer had also given leave for a bottle of beer for each man not a total abstainer; and one would have fancied the patients had been starved for a week to judge by the way the food, especially the baked potatoes, disappeared. A very muscular, broad-shouldered medico had been pressed into service for carving, and his wife, who had come to help hand round the plates, was so bright and cheery to the men that one of them was heard to say afterwards, "She's a real lady, she is." Lots of vegetables and big puddings completed the feast, and by the time cigars had been given out (the gift of an officer) one felt that, although so far from home and friends, our men had been made as happy as possible. I must not forget to add that one Lancer, suffering from a kick from a horse and not allowed diet, was made happy by a promise of some Christmas pudding whenever convalescent.

### In a Cottage Hospital.

SURELY no Christmas is ever so merry as that spent in hospitals, and no one could have had a happier one than we did in 1905 in the small Blackheath and Charlton Cottage Hospital. We have in all twenty beds, with a matron and two staff nurses, one on day duty and one on night, and two probationers. All our beds were full last year, with the exception of one private ward, and eleven out of our nineteen patients were children, the youngest, a cherub of a boy of two and a half, with a cherry-coloured countenance and a fractured leg. During the week before Christmas the fiat had gone forth that no one was to count on having any off-duty time from the Friday before till the Friday after Christmas, as there was so much to be done. Boxes of lovely toys and presents poured in, also holly and ivy from the country, looking so bright, and we were all busy making wreaths. On Saturday we decorated the corridor and the Christmas-tree was dressed in the women's ward. It was a splendid tree, laden with toys and useful presents, and our corridors looked very pretty with festoons, wreaths, and fairy lights. We also put up the fairy lights in the wards, and the gas had soft pink shades, the whole being most effective. Sunday brought rest, and at last Christmas Day arrived. Everyone was told to hang up their stockings on Sunday night, and there was much excitement in the morning on seeing what Father Christmas had brought in the night. We day nurses began by going to church, so we were a little later than usual in the wards that morning. "A merry Christmas" sounded on all sides, and all aches and pains seemed for a short time forgotten. Baby was sitting on the entire contents of his stocking. A train, a gollywog, and a box of chocolates were all mixed up together in the strangest way, with a boy in the midst, the chocolates sticking on to him, and a larger smile than ever on his always smiling face. The ward work did not take long that morning, and when matron came round we had prayers and a Christmas hymn in each ward. After this the tables were laid for dinner in the women's ward, and everyone was able to come down to the meal. From the men's ward at the other end of the hospital, when twelve o'clock arrived so did our senior surgeon, who always carves the turkey on Christmas Day, and we clapped him as he walked into the ward, accompanied by others of our visiting staff. Grace having been said, as soon as he had been pinned into a towel, he proceeded to carve. It was a good

CHRISTMAS DINNER AT A COTTAGE HOSPITAL.

turkey, and so were the pudding and dessert that followed; and there was jelly for those who could not eat pudding, there being one small man at dinner with stitches only taken out the day before, after an abdominal operation, and another little one with a pneumonia crisis barely over in time for Christmas. Crackers came next, and great fun they were; and then we were all asked to stand still while our photographs were taken by one of our visiting staff.

IN A COTTAGE HOSPITAL—*continued.*

After this it was time for everyone to rest, so they were all taken back to bed and the wards restored to their normal condition. "There, nurse, I hope you will enjoy your dinner as much as I have mine," said one to me as I tidied up her bed. "I had five helps," said Tommy on his return to his own ward, "and an orange; that made six." I thought his new red jacket fitted extra well; indeed, all the children reminded me of the little boy who said after tea, "Carry I up, but don't bend I." After this we went to dinner, our turkey being sent as a present by one of our committee ladies for the nursing staff. After dinner we sat down for a short time, as the patients had visitors, and at 4 P.M. we had service. Nearly all were able to go down to service in the women's ward, and nearly all the visitors stayed. We had a short service with Christmas hymns, and an address by our chaplain. After this came tea, and in the evening we sang carols in the wards and spent the evening quietly, everyone being in bed by 8 P.M. As matron said, they had had quite enough for one day, and no one was overtired or cross, or any the worse. I shall bear this in mind, like a good many more things that I have learned since I have been here, and act accordingly when—I am a matron.

And so Christmas Day 1905 came to a close, and I do not think there were any who had not enjoyed themselves. On Boxing Day all our children patients of the past year came to tea in the men's ward, and a real live Father Christmas gave away the presents from the Christmas-tree. On Wednesday the wife of one of our doctors gave a concert in the women's ward; and on Thursday the adult patients of the past year came to tea, and heard a concert in the out-patients' hall, given by the daughter of the chairman of our committee. After all this we were quite ready to settle to our work again, and a little later we nurses went away in turn for a week-end to make up for the hard work we had been through.

### With Californian Gipsies.

It was Christmas Eve, and I was putting a few finishing touches to the new dress I was expecting to wear at the Christmas party to which I was invited. As I did so I could not help congratulating myself that my last case had ended satisfactorily and so opportunely, leaving me free to have a "real good time," as we say out here in California, when a knock sounded on my door.

As I opened it I saw a very uncouth-looking man—half Indian, half Mexican—with three dirty small children, the youngest carried in his arms. I inquired what he wanted.

"Please, mam, my missis is in trouble, an' I come out to fetch you; an' as it's a long way, an' I've wasted time chasin' after doctors who's all out seemingly, you'd best git a move on ye."

I must own my heart sank. It was a cold night for this part of the world, and I felt that I had earned my Christmas treat. I found that the individual had been to four doctors, who had all sent word they were "out"—though I fear I did not have the same implicit belief in that statement as the man had. However, it was evident that, even had I been inclined, I could hardly give the same excuse, so, reluctantly picking up my obstetrical bag and climbing into a rickety vehicle drawn by a mule afflicted with string-halt and a lame white pony, we moved off. The seat was very narrow so I had to hold one little child on my lap, whilst the man drove holding another, the third sitting on my feet.

I did not do much talking as I found that the man was imbued with the idea that his wife was about to do him some personal wrong by bringing into the world another "mouth to feed." He ended up a short conversation on this subject with "It 'ud be jest like her to go an' hev twins!"

I gleaned from him that the event was to take place in an improvised tent on an improvised pallet, and altogether, from what I could gather, the poor creature would lack even the ordinary conveniences for such an occasion.

The caravan had been obliged to come to a halt in the mountains, though when they started nothing had been expected to happen till they got to a town.

We all had to get out and walk occasionally when about to ascend a steeper hill than usual, for the gipsy warned us that "the durned mule would kick the cart into matchwood if urged against its will," and the lame pony was "that cussèd she'd jest stand still, an' the devil hisself couldn't move her if she thought she was about to be put upon."

At last we got to the place, and found the poor woman on a dilapidated cot with four more children near by—the oldest being only about fourteen. They were all huddled round a camp-fire. I speedily had them all put into the gipsy van, and then got together as best I could such articles as I knew would be needed.

The only convenience I could see, but one that I was very thankful for, was a beautiful running stream of water near by, and I quickly had a camp kettle filled and put on the fire.

Two hours later the little Christmas baby arrived—a dark little creature with an abundance of jet-black hair, and with a disposition like its father's, if one could judge by the noise it made, no matter how carefully it was handled.

I was very glad it was not "twins"; but when the time came to wash and dress it, I was reminded of a comic song the chorus of which ends—

"All we've got
Is an old iron pot,
And a frying-pan to wash the baby in"—

for I had to use a utensil for this purpose which had been evidently used to cook the family dinner in. In fact, the father had to empty the boiled haricot beans out of it before he could hand it over to me.

The children in the caravan were evidently conscious that something unusual was going on, for they were all wide awake, and from under the canvas on the wagon their heads would keep appearing, making remarks that were more personal than polite. I took no notice of it till the mother was about to fall asleep, and a pitched battle took place. Then, finding my entreaties for quiet were of no avail, I appealed to the father, who was dozing, and after he had soundly spanked three of them, and promised the others "he'd make them smart on the morrow," we had some peace for a time. The moon was shining overhead, and the only noise was the rippling of the stream over the rocks. Looking at the mother lying peacefully on the roughly made bed, with the white pony and mule tethered near, I could not but think of that Christmas morning when the gentle Holy Mother lay on just such a rough bed, with the little infant Saviour in her arms and, gazing up into the bright starlit sky, I could almost imagine that angels were about to descend with their song of "Glory to God in the highest, and on earth peace, good-will towards men."

When daylight came the man drove off to fetch his sister, who was to take my place.

The two oldest children prepared the midday meal, taking from a box cunningly concealed in the wagon two fat chickens, which, after killing and preparing, were consigned to the gipsy kettle, and which really proved delicious—though my conscience rather pricked me when the little Indian boy, sitting on a rock near by with a "drumstick" in his "paws," confided to me with pride that he "helped his pap git them hens out of a farmer's chicken-house and that they were nearly nabbed" before they succeeded. The potatoes, which probably were procured in the same manner, I refused, in spite of a very pressing invitation—not, I fear, on account of my conscience being too tender, but because they were boiled in the pot in which I had washed the baby!

The Indian returned with his sister soon after, and, like the old woman with the obstinate pig, I got home at last, and "in time to enjoy my Christmas dinner."

### While the Children Slept.

Two A.M. on Christmas Eve: the last sounds, musical and otherwise, of carols, bells, and drunken revelry had died away in the streets below, and only an occasional sound of wheels or the chiming of the near church clock broke the stillness. I was the night nurse in the children's ward, and was mounted aloft on the top of a high pair of steps on the right-hand side of the arch spanning the end of the long ward. At intervals when a few minutes could be spared I had run briskly up them to fasten another of the large white letters in their place, anxious to finish before morning the text which was to surround the arch. I felt pleased with my work. The snowy letters stood out boldly with golden rays radiating from them, having quite the effect of streaks of light. "UNTO US A CHILD IS——" So far my industry had carried me, and then I sat for a minute on the top step to rest, for it was arm-aching work. Below in the little white cots the tiny ones were all sound asleep, dreaming happily of the Christmas-tree in the corner and Santa Claus, who is coming to fill each little sock before morning. Poor mites, in spite of their pain, it is the happiest Christmas Eve many of them have spent in their short lives. All were quiet save one, who tosses and moans continually, unrelieved by all that is done for him; he seems, I think, to fret for someone.

I had taken up the "B" to begin my last word, when the silence of the night was broken by a heavy step on the stone stairs. It sounded like a man's tread, and, surprised, I went quickly to the ward door. Outside stood a big elderly man, very black and dirty, as if with recent work. "I'm sorry to disturb you, nurse," he said, "but the sister said as I might come up for a minute to look at Jim. I wouldn't have come like this, only I'm on the night job, and it will cheer the missis up if I can tell her I've seen the little chap. She'll be crying her eyes out for him to-night." I led the way to the cot of my little restless patient, who started up with a glad cry of "Dadda." When next I passed the cot I stood for a minute to watch the contrast made by the great burly man and the wasted white speck of humanity now sleeping quietly, his hand in the big black one. The father glanced up uneasily at my gaze, and, with almost a sob, began to speak, compelled, it seemed almost against his will, to give vent to his feelings. In disjointed sentences he told me his tale. He and his wife, now getting on in years, had no child, for they had lost their only one years back in infancy, and one cold winter night some two years back, when cleaning out a railway carriage in a siding, he had discovered, under the seat of a third-class carriage, a package. It was pushed well back, and was covered with some dark stuff, so as not to be visible in the half light. He knocked it with his broom, found it was a basket, pulled it out, and on opening it saw, to his utter amazement, a baby fast asleep. He very quickly made known his discovery to the authorities, who, though generally most calm and official, seemed not a little flabbergasted, and exceedingly relieved, "as it was very late," to allow the porter to take it home for the night.

Nothing was ever discovered about the child, and as his wife had taken it to her heart at once, he got permission from the parish Guardians to adopt it.

The child had always been delicate, and a few weeks back his wife had broken her leg and had not been able to get about to look after him properly; and then he got sick, and the doctor said that the only thing was to bring him to the hospital. I listened sympathetically to the sad little tale and then went back to my text. As I finished it, the poor man who had been watching me rose to go, and, looking shyly at my work, said, "Nurse, that do cheer me up. I'm sure to-night Jimmy will come home to us again." And Jimmy did.

### Queen Victoria's Jubilee Institute for Nurses.

HER MAJESTY QUEEN ALEXANDRA has been graciously pleased to approve the appointment of the following to be Queen's Nurses, to date October 1, 1906:—

*England and Wales.*—Florence Collingwood-Greenwood, district training at Bermondsey; Elizabeth Nichol Watson, Birkenhead; Laura Dack, Bloomsbury; Edith Mary Elizabeth Jackson, Bloomsbury; Frances Louise Jones, Bloomsbury; Ethel Mary Pitts-Smith, Bloomsbury; Clara Beatrice Kinnish, Bolton; Margaret Ada Byerley, Brighton; Rosa Isabel Elwin, Brighton; Kate Hunt, Brighton; Nellie Marson, Brighton; Elizabeth Murray Thomson Boyd, Camberwell; Minnie Alice Fowler, Camberwell; Letitia Bowen, Cardiff; Elizabeth Owen, Cardiff; Sarah Jane Lambert, Chelsea; Mabel Jane Lloyd, Chelsea; Jane Mary Jones, Gloucester; Eleanor Bridgeman, Liverpool (Central Home); Bertha Holmes, Liverpool (East Home); Laura Jones, Liverpool (North Home); Mary Agnes Keegan, Manchester (Ardwick Green Home); Alice Rawcliffe, Manchester (Ardwick Green Home); Maria Julia Gaudie, Manchester (Bradford Home); Margaret Jane Cretney, Manchester (Harpurhey Home); Margaret Elizabeth Jones, Manchester (Harpurhey Home); Elizabeth Allen, Manchester (Hulme Home); Florence Susmann, Manchester (Hulme Home); Mary Hensman, Paddington; Edith Kate Roberts, Paddington; Ada Annie Garner, Salford; Lilly Budd, Shoreditch; Hilary Houssemayne du Boulay, Shoreditch; Dorothy Horsley, Shoreditch; Eleanor Jones, Shoreditch; Mary Elizabeth Wallis, Shoreditch; Beatrice Roberson Corbould, Walworth; Edith Alice Morris, Walworth; Ella Geraldine Anderson, Westminster.

*Scotland.*—Isabella Shields Beattie, Isabella Cromarty Dewar, Marion Macphail Fletcher, Deborah Anne Ladbrooke, Helen Stuart Logie, Jessie MacBean, Annabel Campbell Maccormick, Jane MacIntosh, and Catherine MacQuarrie, Scottish District Training Home, Edinburgh.

*Ireland.*—Ellen Mary Kavanagh, St. Lawrence's Home, Dublin; Emily Gertrude Hewitt, Elden Newton, Agnes Mary Park, and Annie Wherry, St. Patrick's Home, Dublin.

### Nursing Exhibition and Conference on Nursing.

An Exhibition and Conference organised by the Provisional Committee of the National Council of Nurses was held at St. George's Hall, Mount Street, London, on Thursday, Friday, and Saturday last week. Almost a third of the room was occupied by the stalls, which, while they formed one of the most attractive features, limited the seating accommodation available. The most interesting stall was that on which nurses' inventions were displayed, and these were distinguished by their ingenuity and simplicity. The miniature steriliser for district work, designed by Miss Böge, is a handy little contrivance, and the smallness of its size and cost (3s.) are both great advantages. Sister Young's telescopic foot-support is a very clever arrangement, and the District Nurses' Bed Rest, an arrangement of pink drill with long straps and a padded back, is useful and easy to make. The hammock chair with umbrella, for consumptive patients, merits commendation; and Nurse George's safe surgical sponges, of a purple colour, are excellent. Nurse Steer's portable bed cradle is an ingenious contrivance. At a stall arranged by Miss Helen Todd was to be seen a capital invention—namely, a spatula lighted at the inner end with a spark of electricity. This was exhibited by Messrs. Allen and Hanburys. "Relics of Barbarism," in the shape of terrible fetters and straps such as were in vogue at Bethlem Hospital in the bad old days, were also on view. At another stall the League of St. John's House Nurses displayed everything needed in the lying-in room. The stalls of firms exhibiting made an excellent show. At Messrs. Allen and Hanburys' there were the "Sister Doris" bed-rest, very light and useful, and a most admirable midwives' case. Messrs. Maw, Son and Sons showed the "Ariston" belt, invented by Sister Beatrice Kent—a very clever contrivance. Messrs. Jeyes had many different preparations of cyllin, the non-toxic bactericide—including cyllin dusting-powder, cyllin lint, and cyllinettes (sanitary towels). Messrs. Garrould had a goodly array, prominent among which was Nurse S. Tonge's midwives' case, a quite new invention which, when closed, forms a table and contains all that is wanted in a very small space. Messrs. Down Bros. showed a large variety of articles, among which was a "Sister Louise" ice cup, and a great number of surgical instruments. Mention must not be omitted of the "Equipoise" bed, couch, and chairs, made by the Equipoise Couch Company, Ashford, Kent, which excited much interest and general approval. The bed can be adjusted to every posture required in surgery and is extremely comfortable. It can be raised or lowered by merely releasing a catch at the side, which the patient himself can do without the least exertion; the bed costs complete 5½ guineas. The couch is made on the same principle and can also be used as a carrying chair. A couch costs 8½ guineas, and one of these was recently ordered by Her Majesty the Queen.

The proceedings were opened on Thursday afternoon, when the members of the Provisional Committee held a reception, at which there was an excellent attendance.

### The Care of the Consumptive.

Miss Isla Stewart, Hon. Vice-President, International Council of Nurses, took the chair at the Conference on Thursday evening, and in the course of a few opening remarks expressed the hope that expert knowledge as to the prevention of tuberculosis would become general in future, instead of expert ignorance such as reigned to-day.

Dr. Kelynack, physician to the Mount Vernon Hospital for Consumption, dealing with the question how nurses could best aid in the tuberculosis campaign, said that every nurse should be a hygiene missionary. The nurse of the future would have to be an educational force for instructing the poor in the prevention of disease. He regretted that nurses showed a distaste for sanatorium life on the ground of its monotony. On the contrary, the study of consumptives opened up a new field of study. It was most desirable that in the out-patient departments of the hospitals nurses should be appointed to visit consumptives in their homes and urge them to take measures of prevention. There was urgent need for havens for dying consumptives.

Miss Helen Todd, matron Royal National Sanatorium for Consumption, Bournemouth, dwelt upon the fact that consumptives of the working classes are only able to stay in a sanatorium for a few weeks on account of lack of funds and because they must get to work as soon as possible. She insisted that ignorance was chiefly responsible for the spread of the disease in families and the community at large; that poor people could not manage to give up employment while the disease was in the early stages; and that they could not afford to remain in the institution when once they were able to do a little work. It was essential that nurses working among consumptives should be able to give convincing reasons for the faith of cleanliness. Unless they were able to do so, the sanatoria tended to increase disease by prolonging the lives of consumptives and making them a source of danger for a longer period. Having shown some flasks in use at the Bournemouth Sanatorium, she concluded by urging that lectures should be given to consumptive patients on hygiene by a doctor whose authority they would recognise.

Dr. Mabel Paine, late resident medical officer, East Anglian Sanatorium, opened the discussion and said that however short a patient's stay was, it might be made educative.

Miss Amy Hughes, general superintendent, Queen Victoria's Jubilee Institute, asked what Queen's Nurses could do with consumptives when confronted with the housing problem, which entailed children sleeping with their parents or at any rate in the same room.

Mrs. Bedford Fenwick declared that they must go back to first causes, and one was that the people of this country had not room to breathe. There was something fundamentally wrong in the distribution of the land. No one should be allowed to have property unless they could keep it in hygienic condition.

Dr. Kelynack said that the enlightenment of the individual was the only remedy for all the problems.

### Nurses' Leagues.

Miss G. M. Rogers, President, Leicester Infirmary Nurses' League, took the chair on Friday afternoon, and Miss Isla Stewart gave a "talk" on Nurses' Leagues. The idea of nurses' leagues, she said, originated in America, where, however, they were called by a grander name. The league at St. Bartholomew's began in 1899 and had a membership now of over 500. Leagues had their use and their pleasure. The pleasure was the making of a meeting-place for nurses who, after their training, were scattered abroad and might otherwise not see each other again. The use was that by means of them organised opinion could be expressed. When leagues had been formed all over the country and affiliated, they would then have a national league, and later on an international league, and then they would be able to keep in touch with nurses all over the world.

Mrs. Bedford Fenwick, referring to leagues for nurses in private nursing institutions, said that the nurses of St. John's House had formed a very successful league. It would be very good for private nurses to be formed into leagues; they were often accused of being the most mercenary part of the profession and needed to be kept up to high professional ideals. In joining leagues nurses should feel that they were going to give something and not to get something. They must be prepared to pay a subscription and help to make their journal a success.

Miss Forrest asked how it was possible to get nurses to take sufficient interest in a league to start one.

Miss Mollett thought that nurses had sufficient patriotism, or *esprit de corps*, to make them want to join anything started by their training school.

Miss Eden told the meeting of a very successful association that had been started in Somerset under the name of the Nurses' Social Union, of which Miss Amy Hughes is President. It kept nurses in the country in touch with each other and with new developments.

Miss Mary Ware asked if there could not be one league formed for all private nurses to join.

Mrs. Bedford Fenwick said that she would be glad to start one for all private nurses in the Metropolis.

### Maternity Nursing.

Dr. Champneys, President of the Central Midwives Board, took the chair at the Conference on Friday evening, and in some prefatory remarks observed that nurses could do either an infinity of good or an infinity of harm, and this applied most of all to maternity nursing. Maternity nurses were left more to their own devices than other nurses, and they had also two lives to take care of instead of one. It had been found that the deterioration of the race was due to bad management of infants at the beginning of their existence. Maternity nurses must be well up in their duties, must know aseptic treatment and have imagination, and above all must be impressed with the importance of their work and be thoroughly conscientious. They had harder work than any other kind of nurses. The qualities of a good maternity nurse could only partly be tested by examination.

Dr. W. J. Gow, obstetric physician to out-patients, St. Mary's Hospital, Paddington, read a very interesting paper, in the course of which he classified the various grades of maternity nurses now at work, and described the training given at Queen Charlotte's Lying-in Hospital. A three-months course was, in his opinion, too short; the five-months course as given at Queen Charlotte's was fairly adequate. A maternity nurse must learn the elements of nursing and how to handle a baby; she should possess a good working knowledge of normal labour, and should know what to do in emergencies when no medical man was at hand. Such knowledge could only be obtained by a course of midwifery; panic was the result of ignorance.

Miss Amy Hughes considered that the attitude of trained nurses towards maternity nursing was a completely wrong one. Trained nurses had become so accustomed to look upon everything from an abnormal point of view that in approaching this question they were inclined to regard every lying-in woman as one in an abnormal state. They did not realise that a woman who gave birth to a healthy child under normal conditions fulfilled a law of nature, and in that sense she was not ill and did not want nursing. Many trained nurses thought that maternity work ought to be left to them, but a good many trainers of midwives did not agree to this. Trained nurses introduced feelings of dread; they were always going to the thermometer or feeling the pulse; they knew too much. Women were wanted who had been trained how to conduct natural labour and to let nature take its course, though they must also know how to act in case of complications requiring the attendance of a medical man. It was a distinct value to a trained nurse to possess a qualification for midwifery, especially in district work, where it was becoming a necessity. One of the problems of the day was mothers ceasing to nurse their babies, and maternity nurses ought to instruct the patients on the wrong they would do their children in such a case. Doctors did not know the tricks that were played by maternity nurses and mothers in order to make them believe the mothers were unable to nurse their children. The old Mother Gamps had sometimes an intimate knowledge of the needs of tiny infants and knew them a great deal better than the modern scientific nurse. Unless nurses loved babies they would never be successful with them.

Dr. W. S. A. Griffith, assistant obstetric physician to St. Bartholomew's Hospital, opened the discussion by remarking that in America the three-years system of training nurses included midwifery training. He would prefer to see the different training of monthly and maternity nurses done away with; all should be trained alike.

Miss Breay thought that matrons of large hospitals should select from the candidates those who had had midwifery training.

Mrs. Bedford Fenwick took the chair at the "talk" on Saturday afternoon, and sketched for her audience the rise and progress of the International Council of Nurses. There would be, she said, an interim Conference of this Council held in Paris next June, and the best way to help French nurses, who had great difficulties to overcome before they could organise, was to visit Paris on that occasion.

Miss M. Mollett, matron of the Royal South Hants Hospital, drew an alluring picture of the many attractions of Paris—social, artistic, and historic.

Miss Edla Wortabet gave an account of the position of French nursing at the present day, dwelling upon the devoted labours of Dr. Bourneville to raise the status of nurses and to educate them. Religious and political questions played a great part in hindering the much-desired improvements, and up to the present it had been found almost impossible to induce women of any but the working classes to become nurses.

### Mental Nursing.

Dr. Robert Jones, President of the Medico-Psychological Association, took the chair at the Conference on Saturday, and said that at the present time 11,000 male and female nurses were working in county and borough asylums, while 1,000 were engaged in private nursing of the insane and 500 in private asylums. There was no work so trying as mental nursing, and in recognition of this, public bodies granted a liberal scheme of pensions for those asylum nurses who stayed the requisite time. Their hours were very long, and they must never look for the gratitude so readily bestowed on other kinds of nurses, as mental patients desired to forget everything connected with their stay in asylums. There were no breaks in the monotony of asylum work, and the record of the time spent by nurses was one of self-abnegation and self-denial.

Dr. G. M. Robertson, medical superintendent, Stirling District Asylum, read a paper which dealt principally with the *entente cordiale* between hospitals and asylums which was gradually being brought about. The progress which had been made in mental nursing was not sufficiently appreciated; still, the fact that an evening out of the present Conference had been set apart for the discussion of mental nursing was a sign of the times. As a result of the good training received by mental nurses there was a great demand for them in private nursing when they left the asylum, partly because they could nurse mental or bodily cases, and partly because they were, on the whole, more prepared to rough it and less trouble in a household. After briefly sketching the course of training of mental nurses, he went on to say that in his opinion there was a great future for hospital nurses who had also gained asylum experience as matrons of asylums. At the present day the matron of nearly every Scottish asylum was a hospital-trained nurse. The results had been most successful, discipline had been much improved, and the asylums generally had become more hospitalised. There was little doubt that in future no nurse would be considered fully trained who had not received the double training.

Miss E. Satchwell, matron, Royal Hospital, Chelsea, in opening the discussion, compared the state of things that reigned in asylums years ago with the present conditions. Skilled nursing was as much needed in asylums as in hospitals. In the latter, if a patient did not obey orders he could be sent out, but not so in asylums. Asylum work afforded scope for the highest intelligence. Women were now playing a much larger part than formerly in the treatment of the insane: they nursed in both male and female wards, male patients being usually much less troublesome than female.

Dr. G. E. Shuttleworth, Hon. Secretary, Asylum Workers' Association, gave a short account of the history and objects of the association and said that he was very glad to hear of the *rapprochement* between hospital and mental nurses, but asked what was to become of male nurses if all the plums were carried off by hospital-trained nurses.

Dr. T. B. Hyslop, Resident Physician, Bethlem Royal Hospital, remarked that at his hospital they took probationers for six months from the large hospitals.

A question was then asked if any general hospitals took mental nurses to train for a year. The reply was in the negative.

Dr. Harding, of Berrywood, wanted to know how hospital nurses got sufficient experience in asylums. There were certain cases which must be nursed by males, and it seemed a great hardship to put hospital nurses of a few months' training over male nurses of experience.

Miss Isla Stewart, alluding to the suggestion of a double training, thought that it was all a question of money, as it would take a very long time.

Dr. Robertson, in reply, emphasised the point that matrons of asylums should play a more important part in the examination of nurses. At Stirling District Asylum the matron gave a course of lectures.

### The Nurses' Hostel and Miss Hulme.

We are glad to be able to announce that Miss Hulme has received from the Board of the Nurses' Hostel Company, Limited, through their solicitors, a letter which is a complete vindication of her character. It is to the following effect:—"Nurses' Hostel and Miss Hulme.—Nov. 22, 1906.—We have the Board's authority to write and say that in superseding your client, Miss Hulme, as superintendent of the Hostel, they did not intend to cast the slightest slur on her character, integrity, or professional ability, and that their action was due to their conviction that Miss Hulme's view of the object of such an institution as a Hostel and its conduct differed from their own." Her only object having, as the result of her firmness and patience, been achieved, Miss Hulme has agreed to forego the action which she contemplated bringing against the Board. She now feels free to devote her attention to the establishment of a hostel for nurses, which she hopes to open about the middle of December. For this purpose, Miss Hulme has secured three houses in Colosseum Terrace, Regent's Park, close to the Park and to several of the chief railway stations, as well as within an easy walk of medical men and nursing agencies. There will, of course, be telephonic communication available night and day, and we do not doubt that Miss Hulme's new hostel will prove popular and attractive, as well as most useful to members of her profession.

### Suicide of a Probationer.

### LETTER TO THE CHAIRMAN OF THE WESTMINSTER HOSPITAL.

No blame attaches to anyone for the sad suicide of Miss Tillett, a probationer at Westminster Hospital, who drowned herself in the Thames on Sunday week evening, her body not being discovered until the following Wednesday. She was wearing the uniform of a nurse. Miss Tillett entered Guy's Hospital about two years ago, and passed successfully through the six weeks' preliminary training. She then went into the wards, but only remained there for a few days, as she suffered from severe and constant headaches, which she apparently thought were caused by the strain of her nursing duties. Accordingly, she gave up her work and went home. As stated by the matron of Westminster Hospital at the inquest, before the East London Coroner on Friday, she subsequently commenced training at that institution, but it may be added that she only arrived there on the 16th of November. Her experience at Westminster Hospital was, therefore, limited to about forty-eight hours. She was placed as an extra probationer in a medical ward, and had no responsible work of any kind. On Sunday, November 18, she was given a four hours' pass, and went off duty at 2 p.m., seemingly in a normal condition. She told the nurse who saw her last that "she was going for a walk and should not be away long." In a letter addressed to the Chairman of the Hospital, Sir John Wolfe Barry, her brother says: "We are perfectly satisfied that my sister received nothing but kindness while at the Hospital and Nurses' Home, and that her death had nothing whatever to do with anything that happened in either." By way of emphasis, he has thoughtfully sent a donation to the hospital. The matron did not know until after Miss Tillett's death that she had been to Guy's Hospital, and she went to Westminster from the Cottage Hospital, High Wycombe, where she had been for two years. She was highly recommended by the matron. In a little diary the unfortunate girl kept while she was at Westminster Hospital, she wrote, "Nurses very nice," a final and pathetic proof that when she committed suicide her mind, as the jury found, was unhinged. It is possible that if she had continued in the tranquil atmosphere of a cottage hospital she might not have lost her reason; but it is only too clear that the excitement of work in a great London hospital was more than she could stand.

### Hospital for Invalid Gentlewomen.

It is a matter for regret that such an excellent institution as the Hospital for Invalid Gentlewomen in Harley Street, founded by Miss Florence Nightingale more than half a century ago, should be hampered by a debt of £600. Moreover, the sum of £6,000 will shortly be needed in order to effect the requisite removal and rehousing of the institution. We hope that the sale of work organised by Miss Tidy, the lady superintendent at the Hospital, and held on Wednesday and Thursday last week, has yielded a substantial sum. It was opened by Lady Maud Wilbraham, and a large number of contributions were forwarded from grateful patients from all parts of the country. Extensive purchases were made by the Duchess of Buccleuch, and the fresh fruit and lovely flowers offered at tempting prices were in great demand. An entire stall was filled by a lady who had begged a trifle of china ware from each of her friends.

### Central Midwives Board.

Meeting in Camerâ.

A special meeting of the Central Midwives Board was held at Caxton House, Westminster, on Thursday last week, for the consideration of the requirements of the Board with respect to training in Poor-law institutions. On the motion of the Chairman, Dr. Champneys, it was decided that the proceedings should take place *in camerâ*, since the nature of the subject forbade its discussion in public. In concurring in this view Mr. Parker Young pointed out that in the ordinary course he was strongly in favour of publicity. This particular matter would, however, come before other bodies at a later date.

### Nurses' Union.

Miss Dashwood invites all nurses able to be present to 5 Cambridge Gate, Regent's Park, on Wednesday next, at 3 p.m., the occasion being the fifth annual sale of work. The sale is in aid of the Nurses' Union bed at Nablus Hospital, Palestine, and the Nurses' Union Native Nurse at Lucknow Memorial Hospital, India. Tea and coffee will be served at 4 p.m., and at 7 there will be short talks on medical work. The Hon. Gertrude Kinnaird will deal with "Peeps at our own Native Nurses at Work at Lucknow"; Mrs. Gibson with "Nursing the Sick in Teheran"; and "Nursing at a Church Missionary Society Hospital in Cairo" will also be discussed.

### Cookery Competitions.

An interesting Cookery and Food Exhibition at the Royal Horticultural Hall, Vincent Square, Westminster, was opened on Tuesday by the Duchess of Albany and remains open until the end of the week. There are, among others, competitions for school children, naval cooks, army cooks, amateurs, and in addition to the fish cooking and the fowl competitions, there is a hospital section. But as it could not be opened until Thursday for lack of room, we are this week unable to do more than express regret that visitors on the first two days had not the opportunity of inspecting it.

### Everybody's Opinion.

### DEGRADING AND CRUEL CHRISTMAS PRESENTS.

"A Poor Penniless Pro" writes: Cannot something be done this Christmas to put down the practice of giving presents to those in authority? Collections are always made for this purpose from those who can ill afford to contribute and given to those who are earning double and treble the salary they earn, yet if you do not give you are looked upon as mean; the result being that home gifts cannot be purchased for lack of funds and girls are in debt for months to come. I am speaking of what I know to be facts. The gift may be only one of flowers, but when it means 1s. towards each present to, say, matron, medical superintendent, two doctors, and ward sisters, it is very hard on the poor pro's. Porters and maids have also to be tipped. Perhaps if all made up their minds not to conform to the practice it could be put down without an appearance of either meanness or ill-will.

*** We invite early information of every proposed subscription to gifts of the kind mentioned in the above letter. No matron, superintendent, sister, or other official can surely countenance or accept Christmas gifts of this character? They constitute a grave scandal, and must be fully exposed if necessary.—Ed. The Hospital.

### MALE NURSES.

"Certified Male Nurse" writes: Having read the reply to the correspondent as to why male nursing had not progressed as it should have done, I think the reason you put forward is unfair. As you state, the only hospitals open to men for training where certificates are granted are the National Hospital and the military hospitals. Male nurses are a decided success at the National Hospital. They have been complimented on their work in the hospital by the medical staff and they are in demand for private work. With regard to the Military Hospital male nurses, I know nothing about them. Probably they are not of the best. I am sure that if one of our large general hospitals in London were to offer to train male nurses, there would be more than sufficient good men go in for the training. Because male nurses were not a success in America, that is no reason why they should not be a success in England. I trust in fairness to those male nurses who are trying to make male nursing a success, that you will find space for this letter.

### A RETROGRESSION IN EDINBURGH.

"One Interested in the Improvement of Mental Nursing" writes: Scotland has not earned the name of being backward in the study and treatment of mental diseases; we have therefore the greater reason to regret the step taken by the recently appointed superintendent of a private mental hospital near Edinburgh. Some eight or ten years ago the ex-medical superintendent of Mavisbank, Limited (Dr. G. R. Wilson), argued that one of the most important factors in mental therapeutics was the proper skilled nursing of the patients by intelligent and well-educated women. Not without strenuous efforts on the part of doctor and matron (for this great branch of nursing does not appeal to the majority) an efficient staff was gathered together, its members drawn from the upper middle-class of Edinburgh and its surrounding districts, principally the daughters of professional men. These efforts to improve mental nursing were amply repaid at Mavisbank by the increased comfort and well-being of the patients, this, indeed, being so marked as to merit frequent recognition from the Visiting Commissioners in Lunacy. Now, however, Mavisbank, Limited, has changed hands. A new medical superintendent reigns; a "go-ahead" fellow, who guarantees to pay the shareholders a reasonable dividend in the year, and who vaunts on his hospital prospectus—"We have now good working nurses." These estimable nurses are drawn from erstwhile ward and kitchenmaids. Good workers they may be, but whether they are able to wrestle successfully with the intricacies of "the mind diseased" leaves room, I think, for grave doubt.

### THE STANDARD OF NURSING.

"Mater" writes: Having read your paper and the letters respecting the standard of nursing I was very pleased to see the reply by "M. G. B.," who must, I am sure, be a very genuine and sensible person. But I think that there are many matrons and others who are of opinion that only ladies should have the privilege of being a nurse. What constitutes a lady—manners or money, or what? Perhaps some of your readers could tell me why it is so difficult for a wardmaid to become a probationer? I have a daughter, now twenty-four years of age, whose ambition for years has been to become a nurse. She has worked in hospitals in all about five years, hoping in time to get in as probationer; yet for months she has replied to advertisements, but with no good result. I think it is most unfair that because a girl has preferred to do the hard work, instead of living a lazy sort of life until she was old enough to train, she should be debarred from training. I am not speaking of her case alone, for at the present time I know of a good faithful nurse, who, I am pleased to say, has got to the top of the tree, although only originally a wardmaid, but it would surprise a great many people to hear what she has to say of the insulting remarks by matrons and others, who, if they had been ladies, would have known better. Why if a "common" Tommy Atkins can become a general, why should not a "common" wardmaid become a nurse?

### Appointments.

[No charge is made for announcements under this head, and we are always glad to receive and publish appointments. The information, to insure accuracy, should be sent from the nurses themselves, and we cannot undertake to correct official announcements which may happen to be inaccurate. It is essential that in all cases the school of training should be given.]

Beckett Hospital, Barnsley.—Miss E. Edmondson has been appointed matron. She was trained at Edinburgh Royal Infirmary, and has since held appointments at several provincial hospitals, at one of which she was assistant matron.

Darlington Fever Hospital.—Miss J. Forsyth has been appointed staff nurse. She was trained at Bethnal Green Infirmary and Huddersfield County Borough Sanatorium. She has since been charge nurse at the City Hospital, Walker Gate, Newcastle-on-Tyne.

East Suffolk and Ipswich Hospital.—Miss E. F. Eyles has been appointed sister. She was trained at the East London Hospital for Children, Shadwell, and Poplar Hospital for Accidents, where she has also been staff nurse.

General Hospital, Niagara Falls, Ontario, Canada.—Miss A. Hayhurst has been appointed lady superintendent. She was trained at Westminster Hospital, and has since served with the Army Nursing Reserve in South Africa.

Hayes Cottage Hospital.—Miss Ellen Hanley has been appointed matron. She was trained at Mile End Poor Law Infirmary, and has since been head nurse, temporary matron, etc., at Isleworth and Shoreditch Poor Law Infirmaries and Uxbridge Cottage Hospital.

Kettering General Hospital.—Miss Lizzie Varley has been appointed sister. She was trained at the District Infirmary, Ashton-under-Lyne. She has since been sister of the children's ward at the West Norfolk and Lynn Hospital, charge nurse at the Brook Hospital, Woolwich, and she has also done sister's holiday duty at the Lancashire and Yorkshire Railway Accident Hospital, Horwich.

Longton Cottage Hospital.—Miss Sarah E. Barlow has been appointed matron. She was trained at the Stanley Hospital, Liverpool, and has since been special nurse at the Jessop Hospital for Women, Sheffield, sister in charge of the children's ward, sister of the operating theatre, and male wards at the Stanley Hospital, Liverpool, head nurse at the Longton Cottage Hospital, and Matron of the Fleetwood Cottage Hospital.

Southern Hospital, Clifford Street, Manchester.—Miss Kate Smith has been appointed staff nurse. She was trained at the General Infirmary, Burton-on-Trent, and has since done private nursing at Leeds.

### Presentations.

Clifton and Bristol Nursing Home.—Miss C. Kershaw, sister of the Clifton and Bristol Nursing Home, 4 Chesterfield Place, Clifton, Bristol, having resigned her appointment after six years' excellent work, the private staff presented her with a gold-mounted Swan pen in silver chatelaine case as a mark of affection. The home staff presented her with a silver-mounted blotter.

### Death in our Ranks.

We regret to announce the death, on November 20, of Miss L. D. Hislop, for some years nurse at the Chelsea Hospital for Women, which took place in a nursing home in Glasgow, where she had undergone an operation.

### Christmas Presents.

### AT PENBERTHY'S.

We shall all soon be launched on the fascinating quest for Christmas gifts, which is apt sometimes to prove rather tantalising. All gift-seekers who yearn for a bewildering variety of choice should turn their steps to Mr. Penberthy's, 388-392 Oxford Street. Gloves are presents that may always

A Welcome Gift.

be relied on as warranted to gain a warm welcome. Suèdes in all colours at 3s. 6d.; fine kid at 3s. 6d.; pique suède at 2s. 6½d.; chevrettes at 1s. 11½d.; then, for hard wear, four-button cape gloves at 1s. 11½d. Some very cosy-looking lined gloves can be had for 2s. 11d., and real gazelle lined at 5s. 6d. Handkerchiefs claim attention next. A special set of four were particularly attractive, all of pure linen with hand-embroidered corners, one with lilies of the valley, one with forget-me-nots and the true lovers' knot, one with shamrocks, and the last and daintiest with thistles. The price is only 1s. 6½d. each. Some with two rows of insertion and lace edging cost 1s. 0½d., and others having a bordering of drawn thread were 10½d. each. A charming little handkerchief with Vandyke border, ornamented with very good imitation Valenciennes, was 1s. 11½d. Some hand-drawn handkerchiefs done by Indians were a distinct novelty, and cost only 1s. 6½d. Silk handkerchiefs with silk Maltese lace were 6s. 11d.—quite exquisite. Handkerchief-cases with half a dozen linen hem-stitched handkerchiefs can be had for 4s. 6d. In the fancy department novelties crowd upon us. Three dainty little blouse-pins for 1s. 6d. Hat-pins in the shape of pansies and thistles at 1s. each. Tasteful calendars in frames at 1s. each, or with gilt top at 1s. 6d. Those charming wallets which have so beautified the indispensable bags are at all prices from 5s. 11d. and in all colours; larger ones capable of holding a fair amount can be had for 12s. 6d. Another very pretty novelty is a neat photo-frame made of beaten copper on oak, with a motto. "Dinna Forget" is on some of those Mr. Penberthy has now in stock, and the price is only 1s. Such frames should find their way into many sitting-rooms and bed-rooms with the face of an absent friend enshrined in each of them.

### AT ANDERSON'S.

Whatever may be the climatic conditions prevailing at Christmas, presents of a mackintosh character will be extremely acceptable. Messrs. Anderson, Anderson and Anderson, Limited), 37 Queen Victoria Street, E.C., are famed for their waterproofs, which can be had at all prices and in all styles. The "Beresford" in white proof, from 21s., is a very useful garment, without the "skimpiness" that so frequently characterises waterproofs. Coats with leg-of-mutton sleeves in green, pinhead coverts can be had from 7s. 6d. to 10s. 6d. each, and plain capes 27 in. to 30 in. from 5s. 9d. to 7s. 11d. each. Travelling rugs, from 5s. 11d., are always useful presents, and so are waterproof holdalls, from 4s. 11d. "Antarctic" overboots at 7s. a pair are most serviceable, especially to the district nurse. The "York" overshoe costs only 2s. 9d. and the "Sandringham" 3s. 3d., and they are a real boon in wet weather. Hot-water bottles are not luxuries but necessities, and can be purchased from

3s. 6d., size 10 in. by 6 in., while a red nikita cover costs no more than 1s. extra. Then there are water beds, usual size, in "super" quality at £9 5s., and in "union" quality at £7 10s., or with central tube at £10 10s.; water pillows from 19s. 6d., and water cushions, or half beds, from 55s. Horseshoe water pillows can be procured at 14s. 6d. each.

### THE NEW GORED APRON.

Nurses whose figures are not "willowy" frequently complain that the fulness of their aprons round the waist enhances the appearance of stoutness. Messrs. Hussey and Co., of Bold Street, Liverpool, are introducing a new apron to remedy this defect. It is so gored that it fits as closely to the hips as a dress skirt, and is remarkably becoming. The two pockets are let into the gored seam at each side, so that they cannot catch on door knobs or the corners of lockers. They are made in three lengths—36 inches, 38 inches, and 40 inches. Those in soft calico cost 2s. 6d., the best Union 3s. 6d., and pure linen 4s. 6d. each. The Union apron should wear splendidly, as it is composed of equal parts of linen and cotton, and washes well, taking a good gloss. The St. Patrick, with square bib, a shilling less in price, is a good, serviceable article, as is the "St. Luke" with a round bib, similar to that which accompanies the gored apron. The caps, collars, and cuffs supplied by this firm are also excellent. Amongst the best are the "Sister Dora" cap, from 10½d. to 1s. 6½d.; the "Ambulance" four-fold collar, 6d. each; and the "Sister Agatha" cuff, which fits perfectly, at 8½d. a pair.

### A DELIGHTFUL EAU-DE-COLOGNE.

There are some women who never seem to care to use perfume, however beautiful it may be. It does not appeal to their sense of pleasure. But the same can never be said of eau-de-Cologne. Well or ill, the fresh invigorating smell of a newly opened bottle is delightful; it is most useful to relieve headache, and a bath in which a few drops of this scent have been sprinkled is most pleasant to tired nerves and tired limbs alike. Those who wish for a really first-class brand cannot do better than ask for "4711 Eau-de-Cologne." It is the purest and best of its kind. The importer, R. J. Reuter, Well Street, Cripplegate, E.C., is just now putting up half-a-dozen bottles in a dainty wooden box for 12s. 6d. It makes a Christmas gift which anyone would be glad to receive and equally glad to use.

### CHIVERS' JELLY.

The child who is not fond of jelly is a "rara avis," and it is always wise in providing an entertainment for children to remember that, whatever else may fail, if there is plenty of jelly to be had, the festivity will be a success. The jellies supplied by Messrs. Chivers—which may be bought at any grocer's—are most easily made and are always delicious to taste. They are flavoured with ripe fruit juices, are pink, yellow, and orange, so as to admit of a pretty admixture of colour, and, instead of being injurious to the smallest child, are distinctly nourishing, cooling, and easy of digestion.

### DELICIOUS CHOCOLATE.

It has been said "When in doubt, play trumps," and equally good advice to those who are uncertain as to what to buy as a Christmas gift, either for a child or a grown-up, might be, "When in doubt, buy chocolate; Fry's by preference." This year there are some very pretty boxes introduced by this firm, varying in price from a few pence to a great many shillings. All purchasers may feel confident that whether they pay a good deal of money or only a very little, the contents will be equally pure, pleasant, and thoroughly wholesome.

### GARROULD'S BAZAAR.

The many visitors to Garrould's Nurses' Saloon should make a point of finding their way to the Christmas bazaar, where they will see all the novelties that the heart of man or woman can desire. A gollywog driving a motor-car, at 9¾d., has only to be noticed to be instantly purchased; and the same may be said of "Ethardo," the marvellous acrobat, who ascends a spiral tower inside a ball, appearing at the top waving flags, 1s. 4½d. "Humpty Dumpty" with a miniature cap over one eye, made in felt at 2s. 11½d., is delightful, and a fur monkey in various sizes, from 9¾d.,

Pomeranian Terrier.—Made in long white hair, 1s. 9½d.; larger size, 3s. 11½d. Also large variety of soft white felt cats and rabbits at 9¾d., 1s. 6½d., 1s. 11½d.

has the strongest claims on the affections. A mechanical toy—the London motor bus, a correct model, but *without* the smell—will enrapture small boys, and is only 9¾d. There is a large variety of bags and wallets; one in roan leather fitted with purse and scent-bottle at 2s. 11½d. is a remarkable bargain. Spectacle-cases to hang on to the waist-band, at 2s. 6½d., are very neat and useful. Purses of all sizes and all prices, all colours and all shapes, muster in great force. Dainty little ostrich-feather fans, at 1s. 0½d., look really charming. Silver photo-frames from 8¾d. are all very pretty, and some novelties in the shape of a pewter stand with calendar, and

The Clifton Funicular Railway.—Amusing toy. The cars are worked by weight, and pass and repass one another, as illustration. 3s. 6d. Also in larger size at 4s. 11d. and 6s. 11d.

another for a bee clock at 10¾d., are very striking. Post-card blocks at 1s. each, and post-cards and calendar combined, at 1s. 2½d., are useful gifts. A neat set of boxes—glove, handkerchief, pencil, string—almost every kind of box one can think of, in light wood, with pretty coloured ornamentation, cost only 10¾d. each. In the same style is a blotting-case. Scissor-cases, containing three pairs of good scissors, cost 3s. 11½d., and there are innumerable travelling ink-pots from 9¾d. Ladies' hair-brushes range from 1s. 11½d., silver initials costing 9d. each and monograms 3s. 6d.

### Notes and Queries.

**REGULATIONS.**

**The Editor is always willing to answer in this column, without any fee, all reasonable questions, as soon as possible.**

**But the following rules must be carefully observed.**

**1. Every communication must be accompanied by the name and address of the writer.**

**2. The question must always bear upon nursing, directly or indirectly.**

**If an answer is required by letter a fee of half-a-crown must be enclosed with the note containing the inquiry.**

*Temporary Work.*

(111) Where can I find employment for a few months, part salary only?—*Maternity Nurse.*

Your best plan is to advertise.

*Buenos Ayres.*

(112) Can you tell me of a hospital in Buenos Ayres where I could get a sister's post?—*Microbe.*

Write to the British Hospital, Calle Perdeiel 74, Buenos Ayres.

*Poor Law.*

(113) Is the Hackney Union Infirmary considered a good training school for nurses?—*Grosvenor Square.*

(114) Is the Sculcoates Union Infirmary, Hull, a recognised training school?—*Chesterfield.*

Yes, both are recognised training schools, but nurses can always obtain such information by writing direct to the matrons of the infirmaries.

*Weak Minded.*

(115) Can you tell me of a home for a weak-minded woman of 30 who can help and pay a little?—*Nurse.*

Write to the National Association for Promoting the Welfare of the Feeble-minded, 73 Denison House, Vauxhall Bridge Road, S.W., for advice. If they are unable to help you, we fear that an asylum is the proper place.

*Where to Train.*

(116) Will you tell me of a general hospital where I can train as a nurse?—*Rudwick.*

Write for "How to Become a Nurse," 2s. 4d. post free, The Scientific Press, 28 Southampton Street, Strand, W.C. There are full particulars concerning all the hospitals which have training schools.

*Medico-Psychological Association.*

(117) How is the Medico-Psychological Association certificate obtained, and what value would it be to a fully-trained nurse?—*Lunatic.*

You must train in an asylum. Write to the Association, 11 Chandos Street, W.C., for full particulars. If you wish to take up mental nursing the certificate is very desirable.

*Maternity Training.*

(118) I am anxious to secure maternity training free of charge.—*Redland.*

Write for advice to the Midwives Institute, 12 Buckingham Street, Strand.

*District Nurse.*

(119) I am a certified midwife. Where can I obtain training for three months or so in a general hospital, and would that qualify me for general as a district nurse?—*Violet.*

Yes. Write to the General Superintendent, The Queen Victoria Jubilee Nursing Institute, 120 Victoria Street, S.W.

*Poor-law Nurses.*

(120) Is the training as good in a poor-law infirmary as in a general hospital? Would a nurse be eligible for posts in general hospitals after such training?—*Inquirer.*

The training is in every way as good in some of the large poor-law infirmaries, but as a rule general hospitals prefer hospital-trained nurses.

*Marylebone Daily Visiting Nursing Association.*

(121) I have been told that this Association has changed its address. Please inform me.—*E. P.*

The address of the Marylebone Daily Visiting Nursing Association is now 45 High Street, Marylebone, and the Secretary is Lady Desart, whose address is 1 Upper Berkeley Street, W.

**Handbooks for Nurses.**

| | Post Free. |
|---|---|
| "A Handbook for Nurses." (Dr. J. K. Watson) ... | 5s. 4d. |
| "Nurses' Pronouncing Dictionary of Medical Terms" | 2s. 0d. |
| "Art of Massage." (Creighton Hale) ... ... ... | 6s. 0d. |
| "Surgical Bandaging and Dressings." (Johnson Smith.) ... | 2s. 0d. |
| "Hints on Tropical Fevers." (Sister Pollard.) ... | 1s. 8d. |

Of all booksellers or of The Scientific Press, Limited, 28 & 29 Southampton Street, Strand, London, W.C.

### For Reading to the Sick.

SAD, YET REJOICING.

He who winter days hath given,
With the snows gives snowdrops birth;
And while Angels sing in heaven,
God hears robins sing on earth.
Only keep thee on the wing,
Music dieth in the dust;
Nothing that but creeps can sing,
All hearts that soar heavenward must.

*E. Rundle Charles.*

In dark days we should look back on bright days and look forward to bright days coming. So, when life is painful we should gather faith and courage, that when God wills to bless us by means of trial we may not be taken unawares, but be strong in Him in whom we have believed. In joy and sorrow alike we must walk on, finding in all that comes to pass help instead of hindrance. The hardest trial for the spiritual life may come when in worldly things all goes well. But in times of loss or care or sickness new temptations come against the soul, needing ever new energy of prayer and will. The goodness of God in happy hours should lead us to repentance, making us more lowly as we count our blessings. In hours of conflict and sadness we should be led to think more of the good gifts which last, and of the nearness of Him who cares for us when we are brought low.

. . . . . . . .

If we are poor in earthly friends there is a Friend who sticketh closer than a brother. His interest in us never changes. His readiness to help and His power are always sure. If we are poor in means and skill to meet the trials of our life, faith can make us rich in all the resources of God. If the cause for which we have in heart left all and in which our life is bound up seems lost, we need not fear. We must do our part, though all we do is, as far as we can see, in vain. God's strength is made perfect in our weakness. When we find out how utterly helpless we are alone, He shows that we are not alone, and shames us for our unbelief.

. . . . . . . .

Our life is safe if it be hid with Christ in God. Nothing can separate us from the love of God which is in Christ. New life and fresh powers of life shall be given as we need. One shall be with us, even the son of God, when we walk in the midst of the fire that tries us. He will keep us from harm. His hand shall check the assaults of evil, making the enemies of our souls serve our true interests. His right hand shall guide our steps and uphold us lest we fall, and strengthen us to go forward till we pass into the light of God's presence, when we shall see Him as He is.—*Anon.*

Save our blessings, Master, save,
From the blight of thankless eye:
Teach us for all joys to crave
Benediction pure and high,
Own them given, endure them gone,

Shrink from their hardening touch, yet prize them won:
Prize them as rich odours, meet
For Love to lavish on His Sacred Feet;—
Prize them as sparkles bright
Of heavenly dew, from yon o'erflowing well of light.

*Keble.*

## Figures and Tables

**Figure f1:**
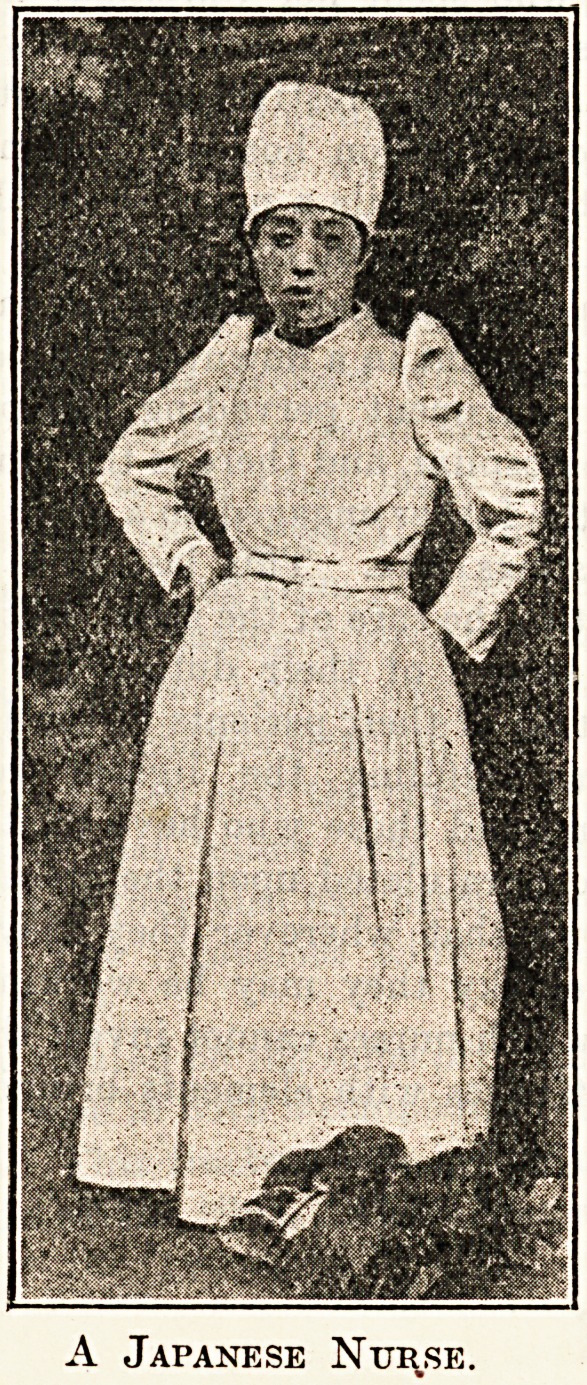


**Figure f2:**
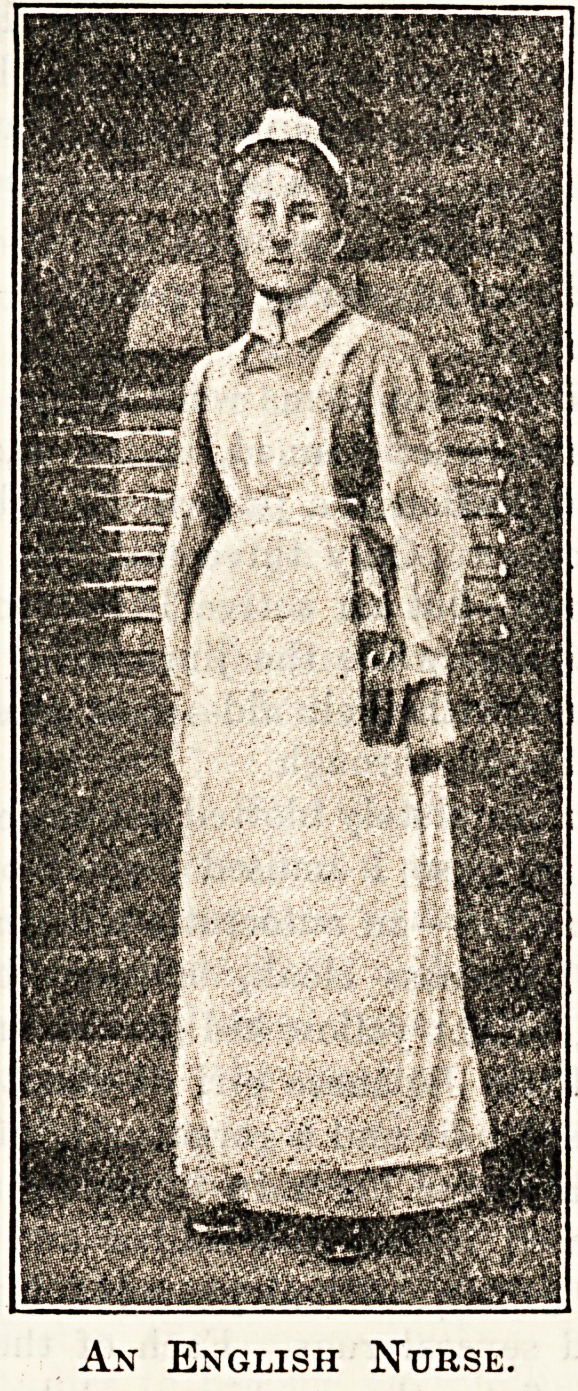


**Figure f3:**
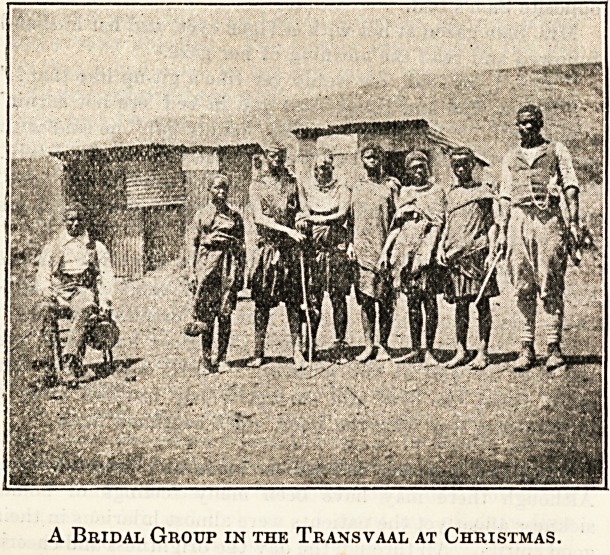


**Figure f4:**
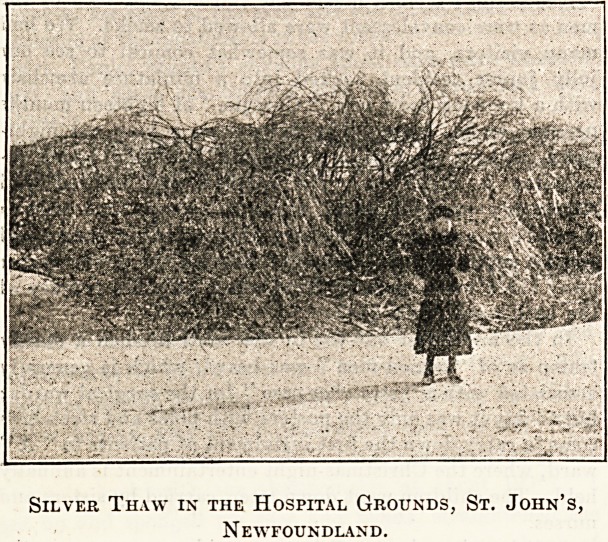


**Figure f5:**
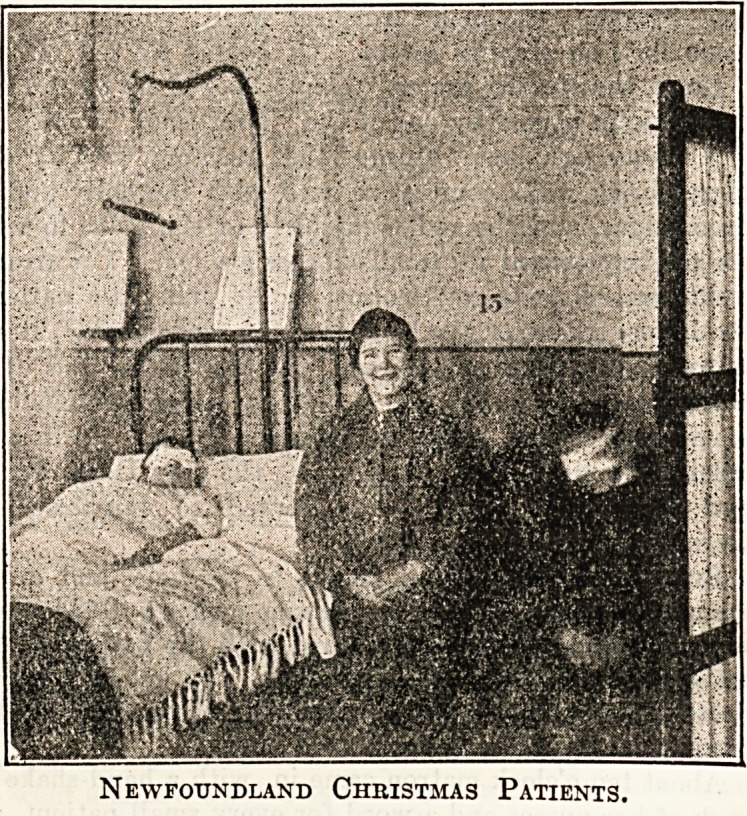


**Figure f6:**
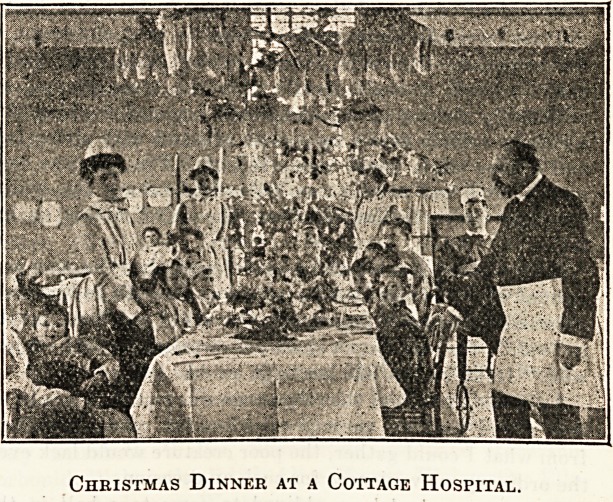


**Figure f7:**
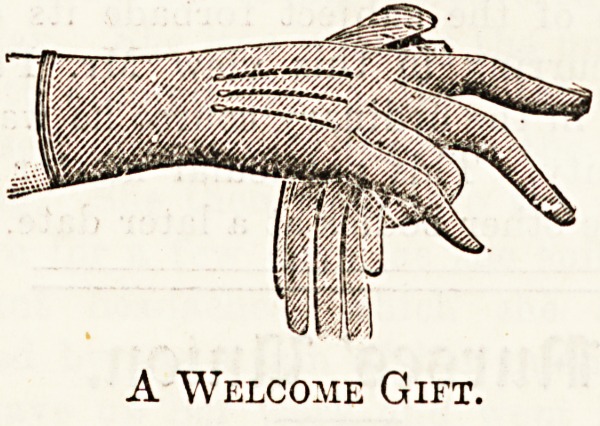


**Figure f8:**
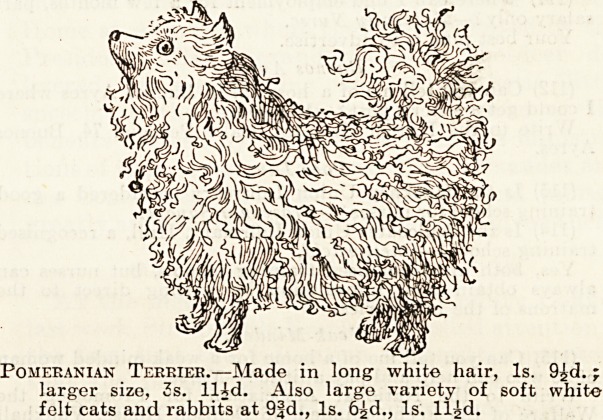


**Figure f9:**